# A comparative petrophysical evaluation of the Abu Roash, Bahariya, and Kharita reservoirs using well-logging data, East El-Fayoum, Egypt

**DOI:** 10.1038/s41598-024-83332-4

**Published:** 2025-01-21

**Authors:** Mohamed Osman Ebraheem, Hamza Ahmed Ibrahim, Ahmed Hosny Senosy

**Affiliations:** 1 Geology Department, Faculty of Science, New Valley University, El-Kharga, 72512 New Valley Egypt; 2 Geology Department, Faculty of Science, Assiut University, Assiut, 71516 Egypt; 3 New Valley University, El-Kharga, 72512 New Valley Egypt

**Keywords:** Well log analysis, Lithology identification, Petrophysical parameters, East El-Fayoum area, Egypt, Environmental sciences, Solid Earth sciences

## Abstract

The exploration and development of hydrocarbon resources in the Western Desert require more continuous activities. The Silah is a newly discovered field in this region. Therefore, this study emphasizes the application of petrophysical evaluation to sandstone and carbonate reservoirs from the late and early Cretaceous. These formations are the most potential hydrocarbon reservoirs in the studied area as a part of the western desert. Additionally, this study involves a comparative evaluation of the Abu Roash, Bahariya, and Kharita reservoirs using well-logging data by applying different cross-plots that are used for determining different petrophysical parameters such as shale volume, porosity, fluid saturation, permeability, and net-to-gross ratio. These logs are gamma-ray (GR), calliper, resistivity (RLA5, RLA3, and RXOZ), photoelectric effect (PEFZ), neutron (APLC), and density (RHOZ). These plots agree with the results deduced from the interpretation of lithologic logs. Fourteen hydrocarbon-bearing zones are identified in the Silah field. Only two zones, namely, Zone 2 in Silah-15 and Zone 1 in South Silah-1X, are considered the best for hydrocarbon generation. These zones are characterized by low to moderate shale volume, moderate to high total porosity, good effective porosity, low water saturation, and high net-to-gross ratio. These zones lie in the Abu Roash/F member. These deduced points prove that the Abu Roash/F member can be an abundant hydrocarbon reservoir. This member in the Silah field appears to be a promising hydrocarbon reservoir because it matches the petrophysical parameters of the investigated zones and others in the northwestern Desert. This suggests that there may be reservoir continuity and similarity.

## Introduction

A well-logging interpretation process is necessary for understanding lithology, petrophysical analysis, fluid saturation, and reserve estimation. In addition, to determine identical facies in drilled wells lacking core data, gamma-ray (GR), spontaneous potential (SP), and calliper logs are used for the correlation of depth and identification of permeable zones^1,2^. A reservoir is characterized by how much fluid it can hold and transfer^3–5^. Therefore, the process of characterizing a reservoir involves determining its parameters and attributes, such as fluid saturation (S), permeability (K), porosity (Φ), and net-to-gross ratio (N/G)^6^. These types of data, separately or in combinations, are needed throughout the productive life of any reservoir if the employment of sound production practices is to be assured. During the development of a reservoir, the data are used to establish spacing and completion intervals of wells and to guide drilling practices^7^.

Since the most easily accessible oil reserves have already been explored and developed, the search for new resources has shifted to more challenging sedimentary basins, making the exploration and development of new oil and gas resources crucial tasks in the petroleum industry. Finding new commercial hydrocarbon discoveries in the shortest amount of time and at the lowest possible cost is the ultimate goal of petroleum exploration campaigns. The abundance of oil and gas discoveries has led to the northern Western Desert being considered a major petroleum province in Egypt^8,9^. Despite continuous exploration activities, many promising areas still undiscovered in the Western Desert exist^10^. These areas need detailed examination and reliable drilling tests^11,12^.

**Fig. 1:**
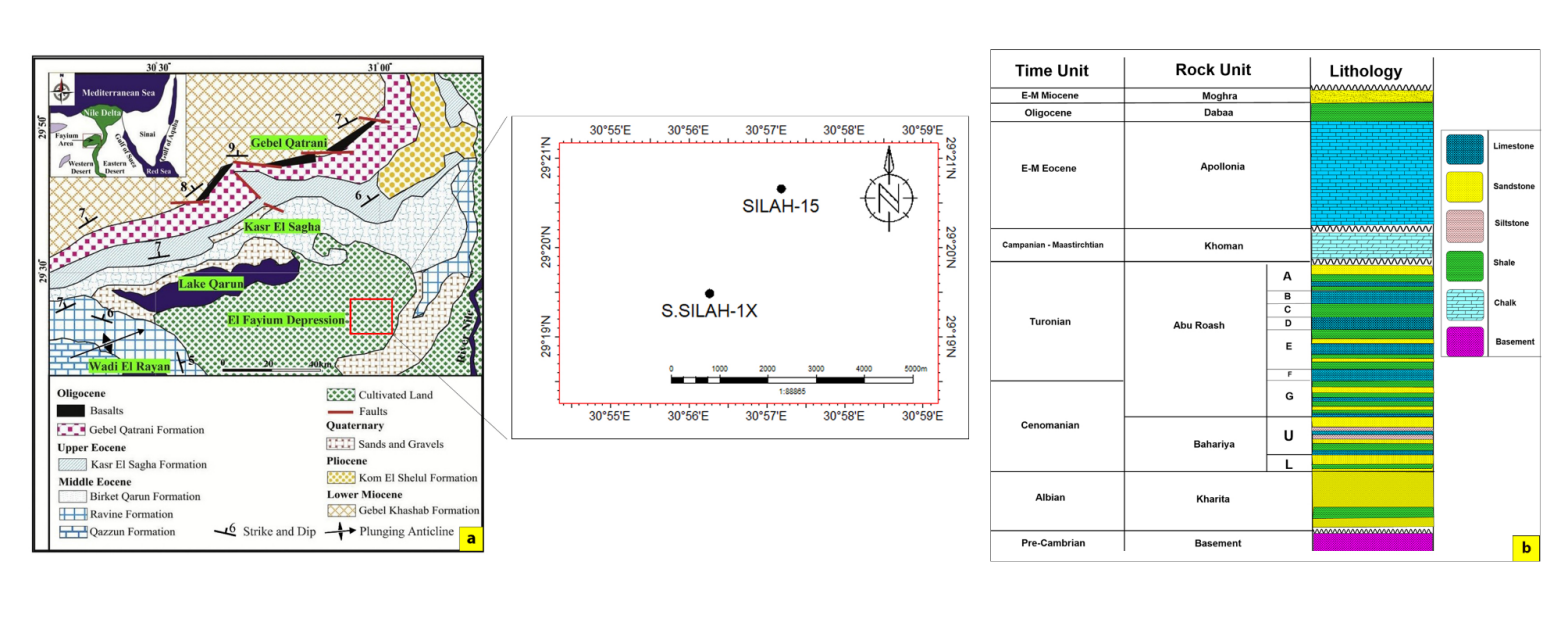
A surface geological map of El-Fayoum (Adapted with permission from ref.13 by Copyright © 2019 Elsevier Ltd. Clearance Center) including the study area (red square) (**a**) and a lithostratigraphic columnar section showing the chronological record of the sedimentary rocks in El-Gindi Basin (was created by using Surfer software, Version 13.6 https://www.goldensoftware.com/surfer/) (**b**).

El-Fayoum Concession occupies a surface area of approximately 9500 km^2^ and is situated north of the Beni Suef Basin and south of the Kattanyia High^13^. This concession area is located near the regional energy infrastructure in the abundant and inexpensive Western Desert. The Silah is considered a newly discovered oil field south of El-Fayoum concession and covers an area of approximately 84 km^2^ (Fig. 1a). The potential reservoirs in this field are made up of sandstone and carbonate, which are present in the Cretaceous formations (Abu Roash, Bahariya, and Kharita).

The present study is concentrated on the early and late Cretaceous formations because most potential hydrocarbon reservoirs and products are present in the sandstone and carbonate within these formations. This study aims to evaluate the hydrocarbon potential of the Abu Roash, Bahariya, and Kharita formations in El-Fayoum. This is achieved through the following steps: (1) estimating different petrophysical parameters from the interpretation of well-logging data, (2) distinguishing between permeable and nonpermeable zones, (3) constructing different cross-plots for identification of the hydrocarbon potential in the studied zones, and (4) recognizing the best hydrocarbon zones in these formations.

## Geological setting

The Western Desert of Egypt is a huge platform consisting of thick sedimentary rocks affected by different tectonic movements with a slight northward regional slope and dip^14^. The majority of the northwestern Desert formed a platform, covered with Neogene sediments, excepting the Abu Roash complex, which is characterized by relatively moderate subsidence near active subsiding basins or depocenter^14^. It includes a chain of NE-to-ENE orientated rift-related basins. El-Fayoum depression lies in the Western Desert of Egypt close to the Nile Valley^15^. Palaeogene sedimentary rocks crop out widely in the depressions^16^.

The surface geology of El-Fayoum is characterized by a vast tract of Miocene deposits that are graded southwards, immediately to the north of Birket Qarun, into a narrow strip of Oligocene sand and gravel at the contact with the underlying Eocene strata^13^ (Fig. 1a). Farther south, the Eocene carbonate sediments are well exposed and cover the Wadi El-Rayan area^17^. Along the banks of the Nile River, Pliocene shale and Pleistocene sandstone and gravel extend through limited outcrops. The basement rocks are unconformably overlain by a similar sedimentary succession that spans the Palaeozoic to Miocene in El-Fayoum region and adjacent Abu Gharadiq Basin to the northwest^18^.

Stratigraphically, El-Fayoum region is subdivided into four lithostratigraphic provinces from north to south: Kattanyia, El-Sagha, Wadi El-Rayan, and El-Gindi (Fig. 1a). Abu Roash, Bahariya, and Kharita are considered economic formations for petroleum accumulation in the Northwestern Desert^17^. The Abu Roash Formation formed during the Turonian–Coniacian period, overlying the Bahariya Formation of the Cenomanian and underlying the Khoman Formation of the Santonian–Maastrichtian (Fig. 1b). This formation consists of seven members, with limestone being the predominant component of members B, D, and F, which were deposited during high sea-level periods. However, clastic units A, C, E, and G were deposited during periods of low sea level^18^. The Bahariya Formation is composed of interbedded siltstone and sandstone with minor intercalations of shale and limestone beds. The Kharita Formation nonconformably overlies the basement rocks and consists of a lower section of siltstone with minor shale interbeds, while the upper section consists of sandstone with minor siltstone interbeds^19^.

## Research methodologies

**Fig. 2:**
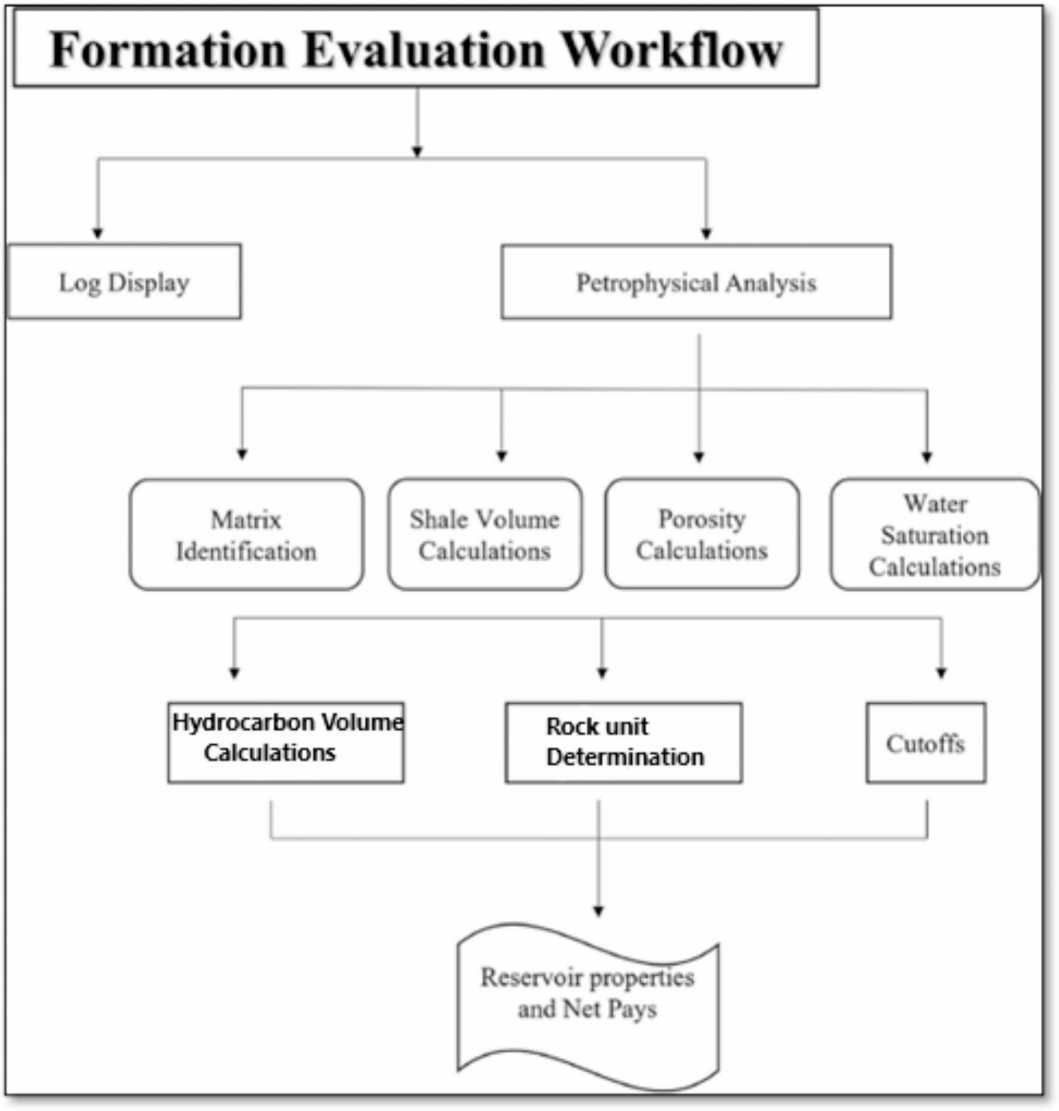
A flow chart showing formation evaluation using well-logging data in Silah.

The wireline logging data for the investigated area were obtained from the Petorsilah Petroleum Company. Two vertical wells (onshore), Silah-15 and South Silah-1X, with their composite logs are available (Fig. 1a). These logs include gamma ray (GR), spontaneous potential (SP), calliper (CL), bite size (BZ), density (RHOZ), neutron (APLC), photoelectric effect (PEE), deep resistivity (RLA5), shallow resistivity (RAL1), medium resistivity (RLA3), micro resistivity (RXOZ), and sonic logs (Table 1). Techlog-version 2015.1^20^ and Petrel-version 2017^21^ software compute petrophysical parameters to deliver a more realistic and accurate formation evaluation (Fig. 2). These parameters are determined by applying the following equations:

**Table 1:** Available Wireline Logs and Kelly bushing of the studied drilled wells.

Well	Available well logs (las file)	KB (Ft)
Silah-15	GR, Caliper, BSZ, SP, RHOZ, APLC, PE, DTCO, DTSM, RLA1, RLA3, K, U, Th, RLA5, RXOZ, and MSF	75
South Silah-1X	GR, Caliper, BSZ, SP, RHOZ, APLC, PE, DTCO, DTSM, RLA1, RLA3, RLA5, RXOZ, and MSF	98

### Shale volume (Vsh)

In this study, the shale volume is estimated using gamma-ray logs via the following Eq. 2^2^:

1 $${\text{I}}_{{{\text{GR}}}} = \left( {{\text{GR}}_{{{\text{log}}}} - {\text{GR}}_{{{\text{min}}}} } \right)/\left( {{\text{GR}}_{{{\text{max}}}} - {\text{ GR}}_{{{\text{min}}}} } \right)$$

where I_GR_= gamma ray index, GR_log_= gamma- ray reading of the formation, G_Rmin_= minimum gamma- ray, maximum density log reading, and I_GR_= V_sh_ in the linear model.

The shale volume was then calculated via the following formula: Shale Volume^22^- Old Rocks, who proposed an equation for shale volume calculation for rocks older than the Cenozoic Era, expressed as follows:

2 $${\text{V}}_{{{\text{sh}}}} = 0.{\text{33}}\left( {{\text{22}}*{\text{ I}}_{{{\text{GR}}}} - {\text{1}}} \right)$$

### Porosity

In this study, porosity was calculated from density‒neutron combination logs^3^.

- Total porosity
  The equation used to compute the total porosity from neutron and density logs may be expressed as follows^23^:
  3 $$\Phi _{{{\text{tot}}}} = \left( {\Phi _{{\text{N}}} + {\text{ }}\Phi _{{\text{D}}} } \right)/{\text{2}}$$
  4 $$\Phi _{{\text{D}}} = {\text{ }}\left( {\rho _{{{\text{ma}}}} - {\text{ }}\rho _{{\text{b}}} } \right)/\left( {\rho _{{{\text{ma}}}} - {\text{ }}\rho _{{\text{f}}} } \right)$$
  where ρ_b_: is the bulk density, which includes both fluid and rock, (read directly from the log), ρ_f_: is the density of the saturating fluid, ρ_ma_: is the density of the rock matrix, and φ: is the porosity.
- Effective porosity
  The equation used to compute effective porosity may be expressed as^23^:
  5 $$\emptyset _{{{\text{eff}}}} = \emptyset _{{\text{T}}} - {\text{ }}[\emptyset _{{{\text{sh}}}} * {\text{V}}_{{{\text{sh}}}} ]$$
  where ∅_eff_ = effective porosity, ∅_T_ = total porosity, ∅_sh_ = porosity reading in a shale zone, and V_sh_ = shale volume.

### Water saturation

In this study, Archie’s model is used to calculate the water saturation of the reservoir rocks. These two models used in the water saturation calculation, yield the best results:

- The water saturation from the Indonesian model is given by the following formula^24^:
  6 $${\text{SW}} = \left\{ {\left[ {\left( {\frac{{VSH^{{2 - VSH}} }}{{Rsh}}} \right)^{{1/2}} + \left( {\frac{{\varphi e^{m} }}{{Rw}}} \right)^{{1/2}} } \right]^{2} {\text{Rt}}} \right\}^{{1/n}}$$
  where a = tortuosity exponent, V_sh_ = shale volume, R_T_ = deep resistivity log reading, R_sh_ = resistivity of the adjacent shale, Φ_e_ = effective porosity, m = cementation exponent, and R_w_ = water resistivity at formation temperature.
- The water saturation from the dual water model is given by the following formula^25^:
  7 $$\frac{a}{{Rt*\phi t^{m} }} = \frac{1}{{Rw}}*Sw^{n} *\left( {\frac{{\phi tsh*Vsh}}{{\phi t}}} \right)*\left( {\frac{1}{{\phi tsh^{2} * Rsh}} - \frac{1}{{Rw}}} \right)*Sw^{{\left( {n - 1} \right)}}$$
  where Φ_e_ = effective porosity that excludes the shale effect: a = tortuosity exponent, V_sh_ = shale volume, R_T_ = deep resistivity log reading, R_sh_ = resistivity of the adjacent shale, Φ_e_ = effective porosity, m = cementation exponent, R_w_ = water resistivity at formation temperature, Φ_tsh_ = total shale porosity, Φt = total porosity, and R_sh_ = shale resistivity.

### Bulk water volume

The BVW is determined via the Eq. 2^6^:

8 $${\text{BVW}} = \Phi *{\text{S}}_{{\text{w}}}$$

### Hydrocarbon saturation

The percentage of S_h_ hydrocarbon saturation (uninvaded zone) is given as^27^:

9 $${\text{S}}_{{\text{h}}} = {\text{ }}\left( {{\text{1}}00{\text{ }} - {\text{ S}}_{{\text{w}}} } \right)\%$$

### Hydrocarbon movability index

The hydrocarbon movability index is a ratio of the uninvaded zone to the flushed zone and is estimated via the following Eq. 2^7^:

10 $${\text{Hc}}_{{{\text{mov}} \cdot {\text{ index}}}} = {\text{ S}}_{{\text{W}}} /{\text{S}}_{{{\text{ox}}}}$$

Hc_mov. index_ = S_W_/ S_ox_ (10).

where S_ox_= water saturation of the flushed zone, which is calculated from the following equation:

11 $${\text{S}}_{{{\text{xon}}}} = {\text{ }}\left( {{\text{a }}*{\text{ R}}_{{{\text{mf}}}} } \right)/(\varphi _{{\text{m}}} * {\text{R}}_{{{\text{xo}}}} )$$

S_xon_ = (a * R_mf_)/ / (φ_m_ ∗ R_xo_) (11).

where n is the saturation exponent, R_mf_ =is the mud filtrate resistivity at the formation temperature, φ is the porosity, m is the cementation exponent, and R_xo_ =is the resistivity of the flushed zone.

## Permeability

In this study, the Timur method is used for the permeability calculation^25^. The empirical estimates of permeability have uncertainties, but Timur has the lowest uncertainty.

12 $$k = \left( {93 \times \frac{{\varphi ^{{2,2}} }}{{{\text{Swirr}}}}} \right)^{2}$$

where K = absolute permeability for oil, Φ_e_ = effective porosity, and S_wi_ = irreducible water saturation of the formation of interest.

### Net-to-gross ratio (h/H)

The net reservoir thickness (h) can be calculated by using the following formula^3^:

13 $${\text{h }} = {\text{ H}}{-}{\text{h}}_{{{\text{shale}}}}$$

where H = the gross reservoir thickness, h_shale_ = the thickness of the shale and Net/ Gross = h/H.

### Net pay

The summary to obtain net pay is a shale volume cutoff of 45%. To define the water saturation S_w_, a cutoff value of 60% was used to define pay.

## Results and discussion

Well-logging data are employed in the analyses and interpretation of the lithostratigraphic sequences^28^. These data are used to construct different cross-plots and compute different petrophysical parameters. This is an essential stage for detecting potential hydrocarbon reservoir zones and determining their potential for the studied drilled wells in the Silah field.

**Fig. 3:**
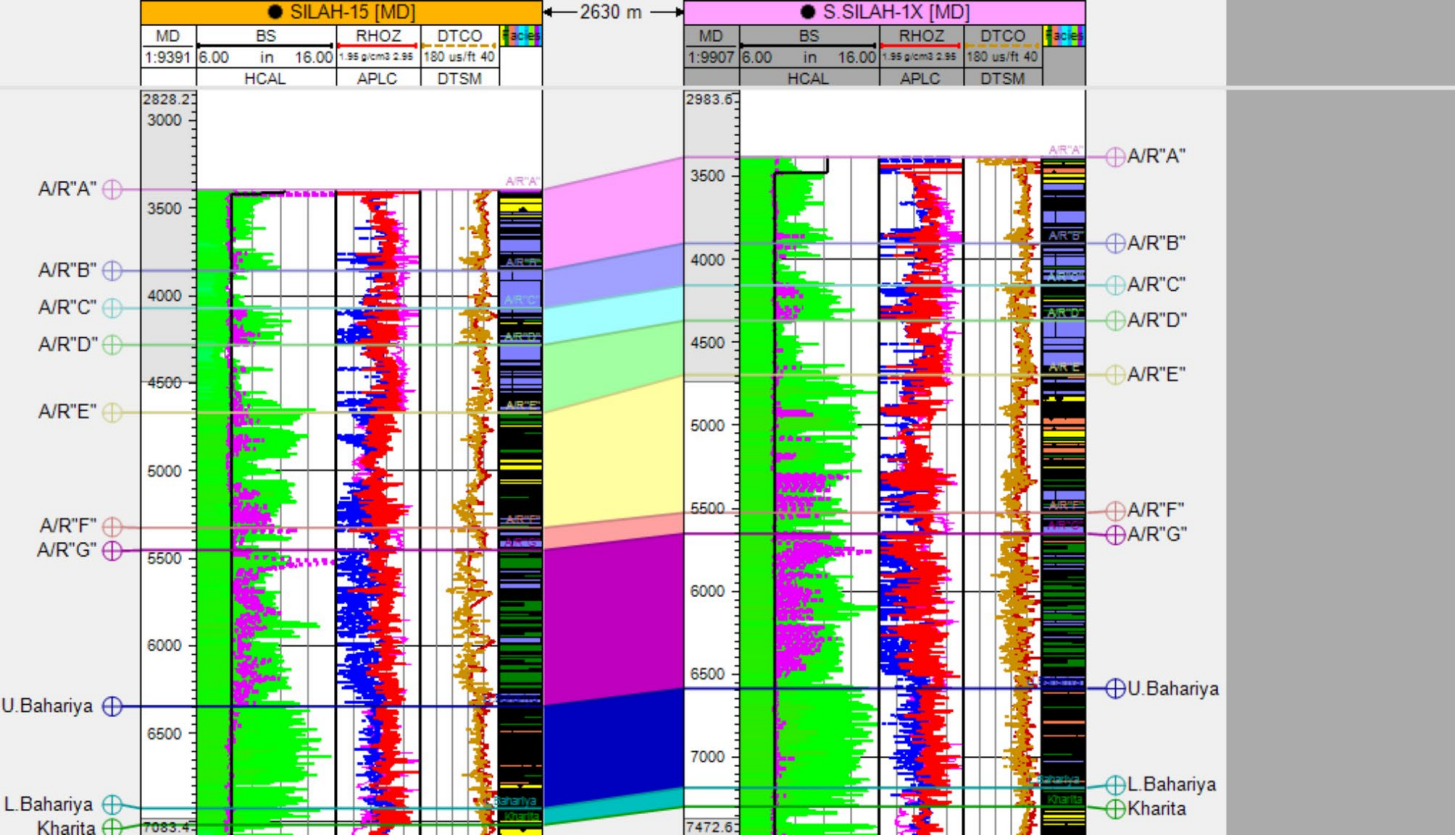
A correlation chart of the studied formation in N-S direction.

**Fig. 4:**
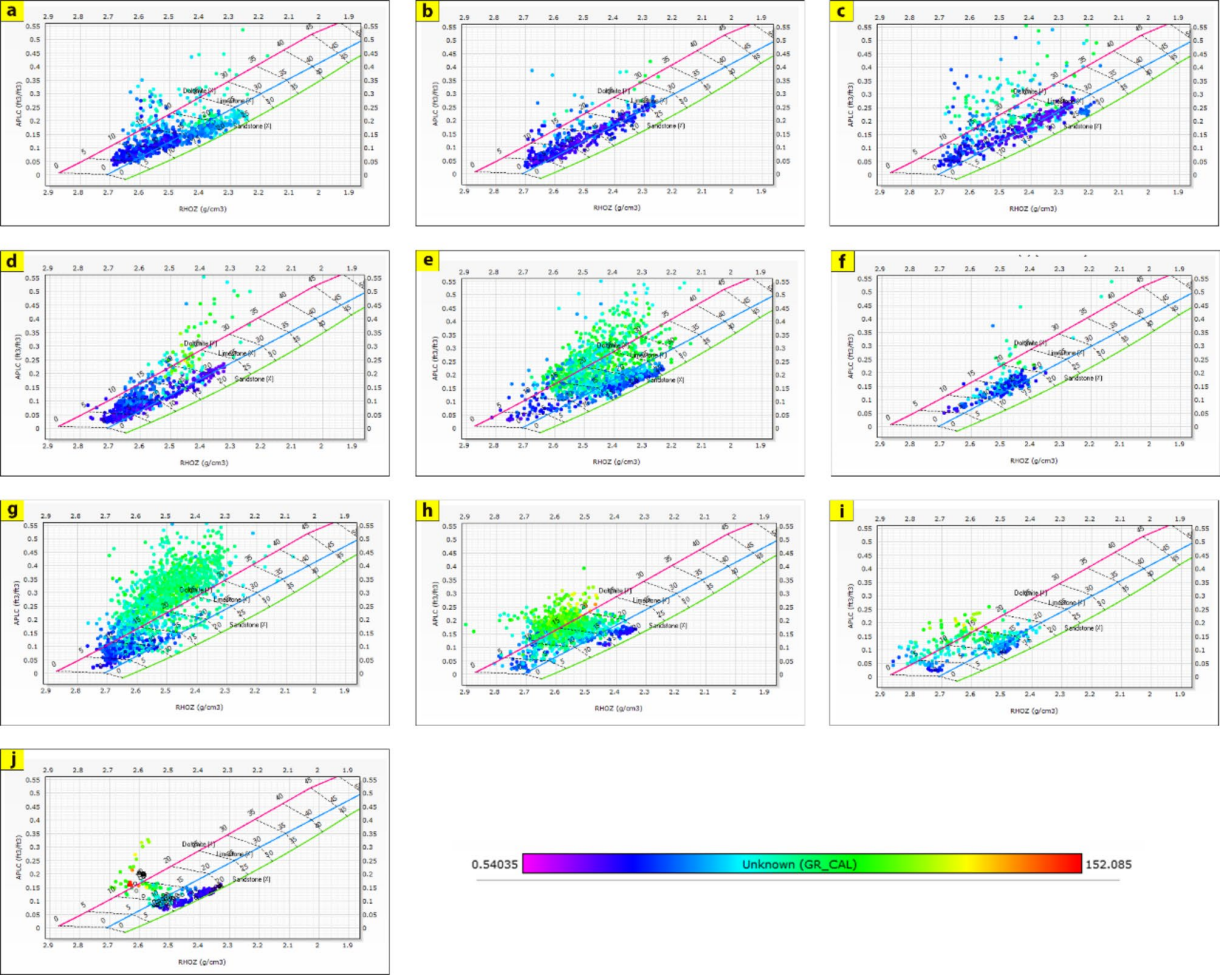
Density-Neutron-Gamma Ray crossplots for; Abu Roash (**a**–**g**), upper Bahariya (**h**), lower Bahariya (**i**) and Kharita (**j**) Formations in Silah-15.

**Fig. 5:**
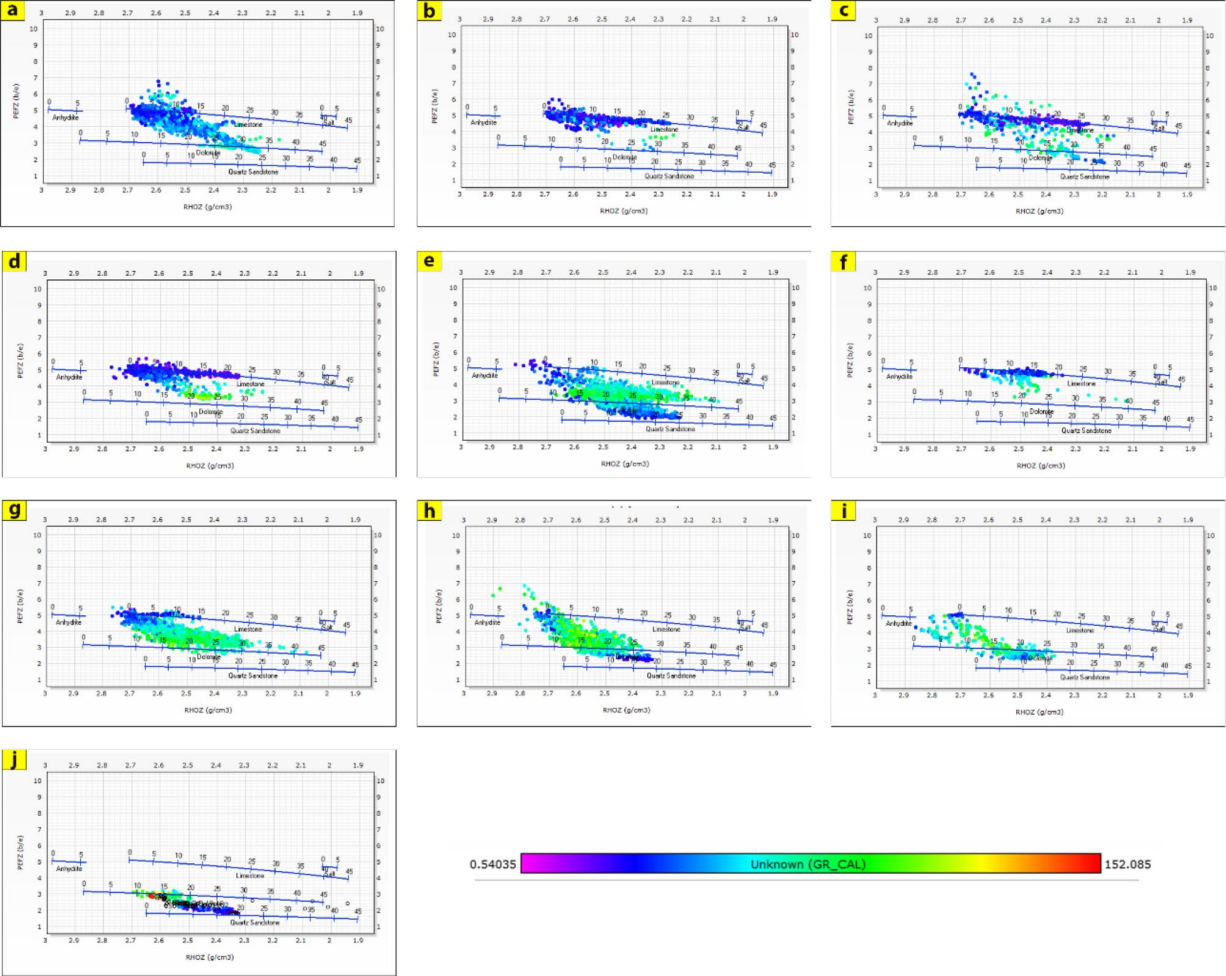
Density-Photoelectric effect-Gamma Ray crossplots for; Abu Roash (**a**–**g**), upper Bahariya (**h**), lower Bahariya (**i**) and Kharita (**j**) Formations in Silah-15.

**Fig. 6:**
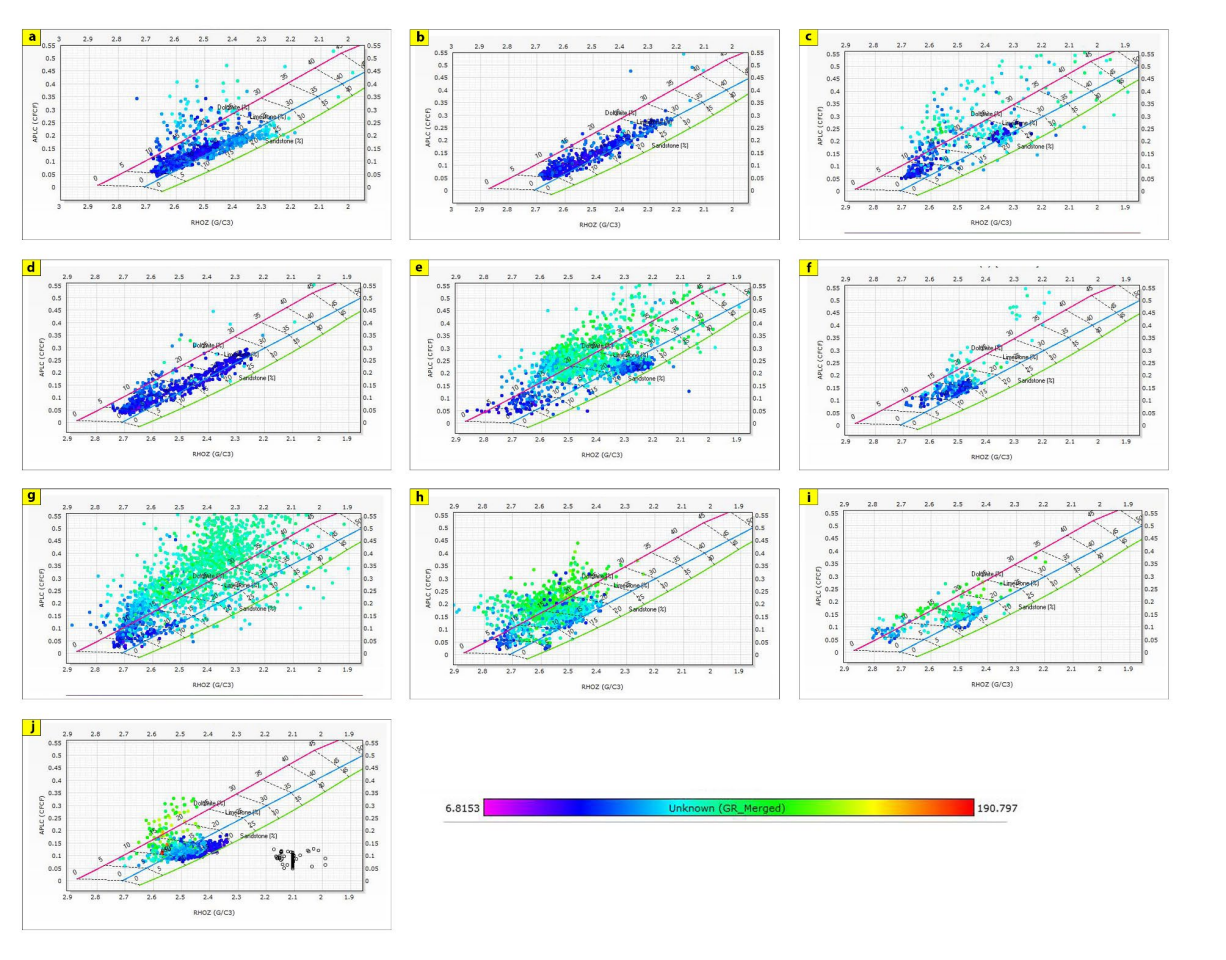
Density-Neutron-Gamma Ray Crossplots of Abu Roash (**a**–**g**), upper Bahariya (**h**), lower Bahariya (**i**), and Kharita (**j**) formations in South Silah-1X.

**Fig. 7:**
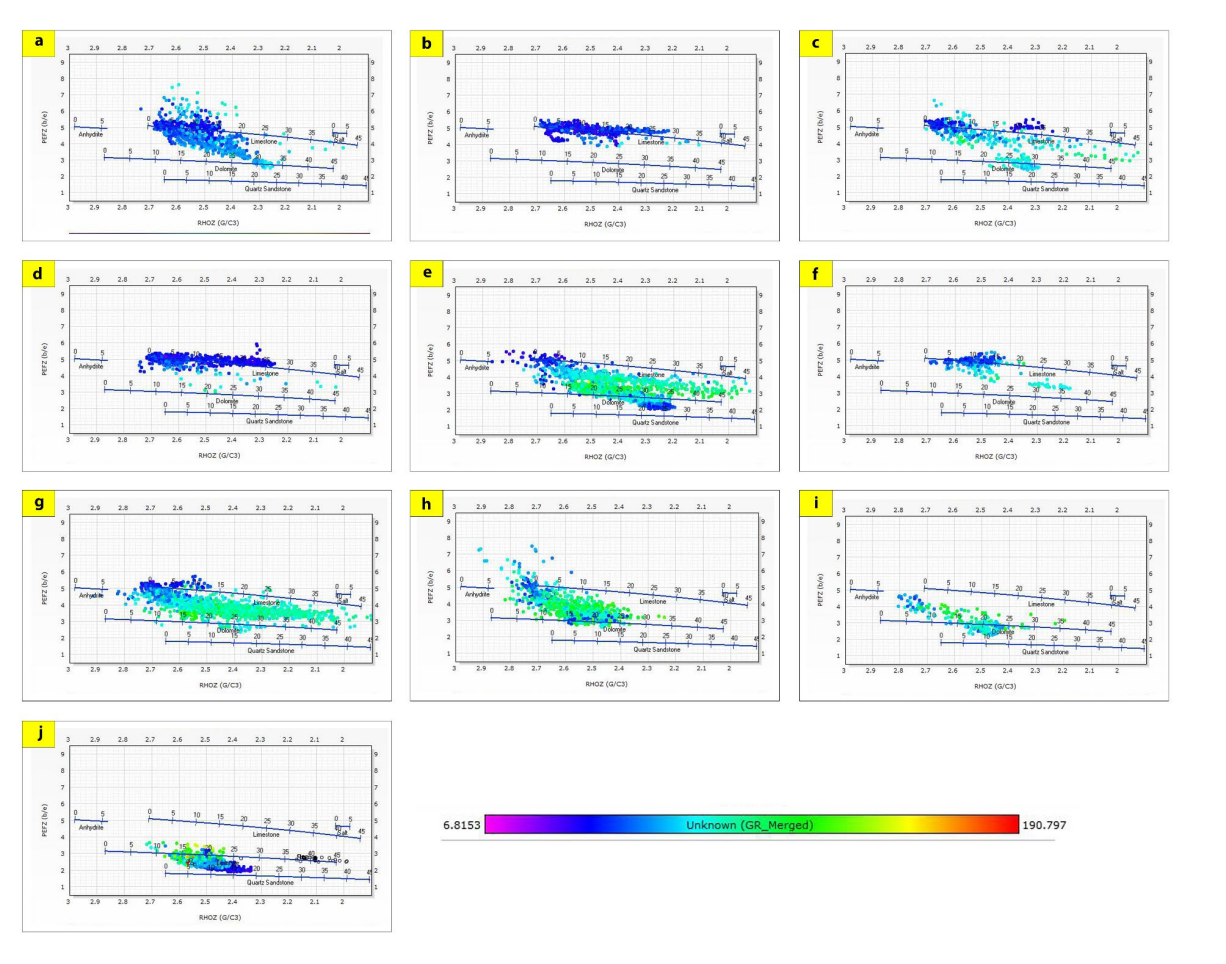
Density-Neutron-Gamma Ray Crossplots of Abu Roash (**a**–**g**), upper Bahariya (**h**), lower Bahariya (**i**), and Kharita, (**j**) formations in South Silah-1X.

The generated lithological logs illustrate the distributions of different lithofacies through the studied wells. These logs reveal the presence of four main types of lithologies: sandstone, shale, limestone, and siltstone (Fig. 3). Cross-plots, especially density-neutron-gamma and density-photoelectric effect-gamma ray plots, identify the porosities and main lithologies, as well as the type and distribution of the shale in the formations (Figs. 4, 5, 6 and 7). These plots show that the main lithologies across the drilled wells are composed mainly of sandstone, carbonate (limestone and dolomite), shale, and siltstone interbedding, which conform to those deduced from lithologic logs. These inferred lithologies are present in Abu Roash A, C, E, G, and Upper Baharyia, but in Abu Roash B, F, and D are made up of mainly limestone interbedded with shale; otherwise, the lower Bahariya and Kharita Formations are made up of sandstone interbred with shale.

**Fig. 8:**
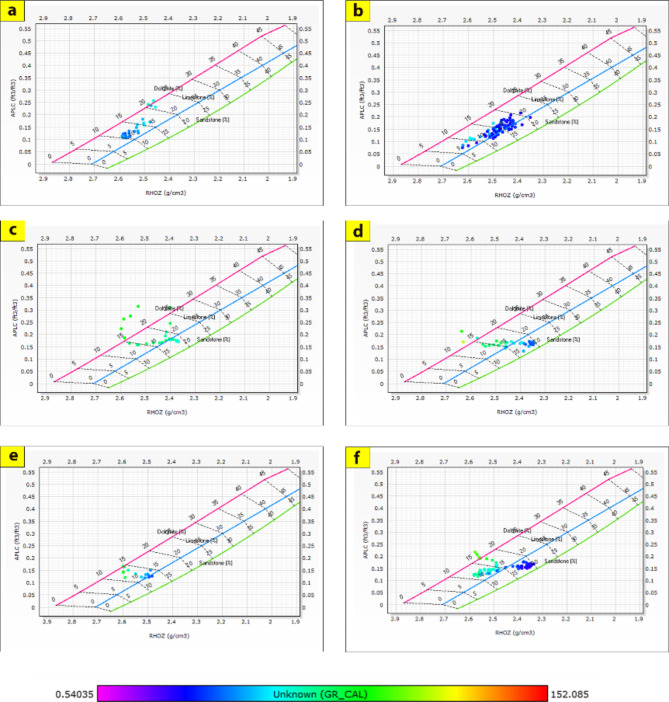
Density-Neutron-Gamma Ray crossplots of the hydrocarbon-bearing zones in Silah-15.

**Fig. 9:**
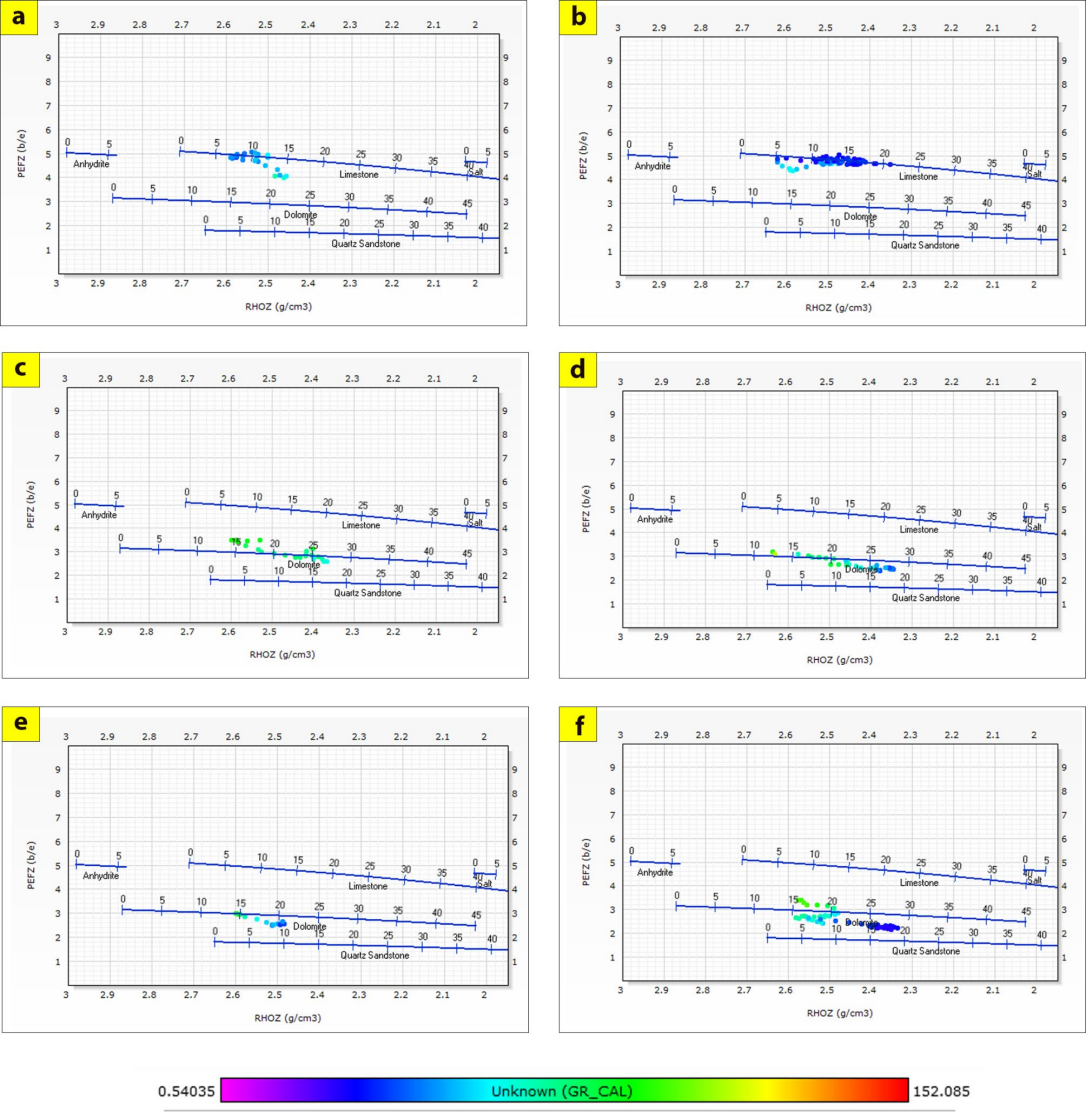
Density-PEE-Gamma Ray crossplots of the hydrocarbon-bearing zone in Silah-15.

**Fig. 10:**
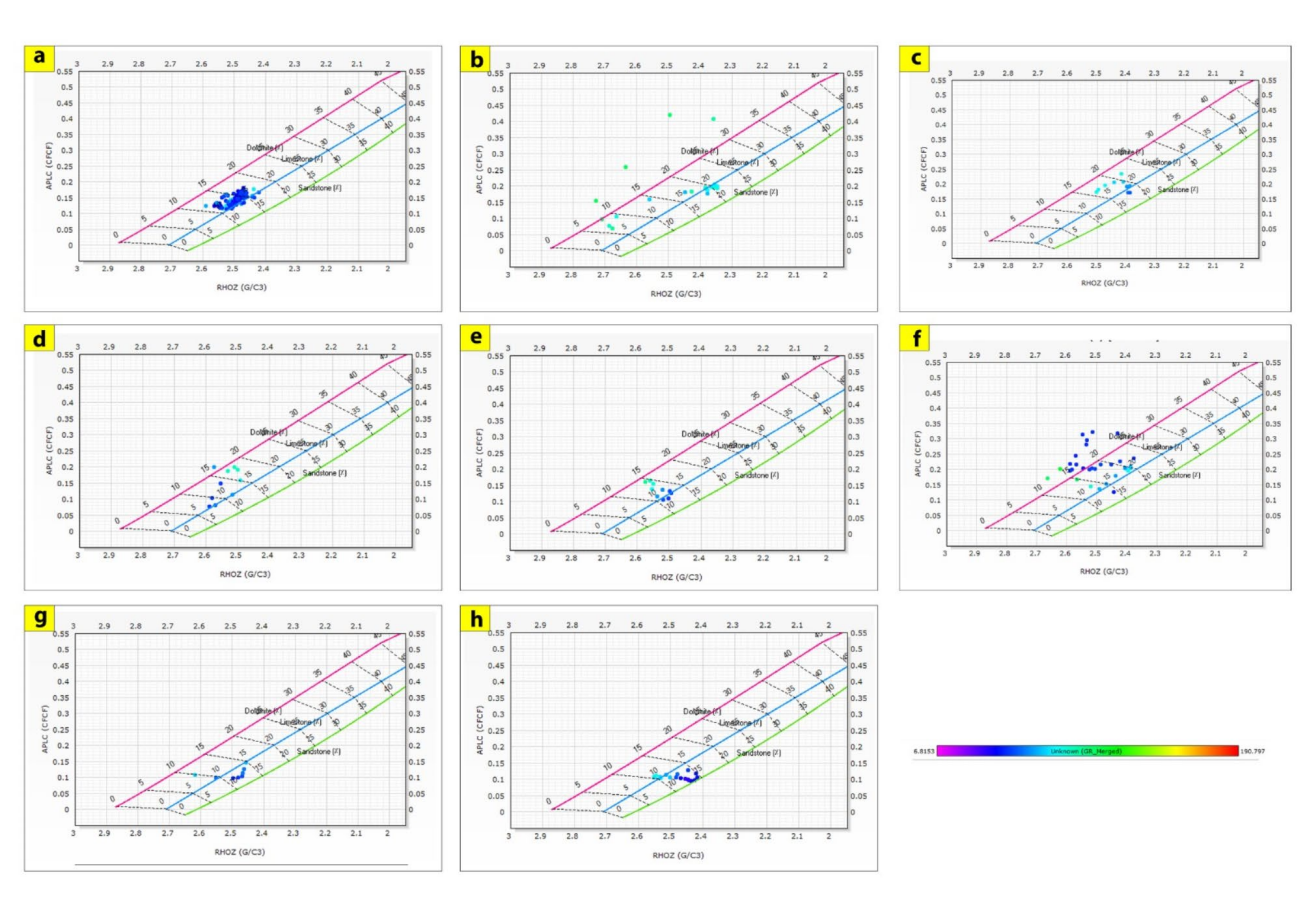
Density-Neutron-Gamma Ray Crossplots of the hydrocarbon-bearing zones in South Silah-1X.

**Fig. 11:**
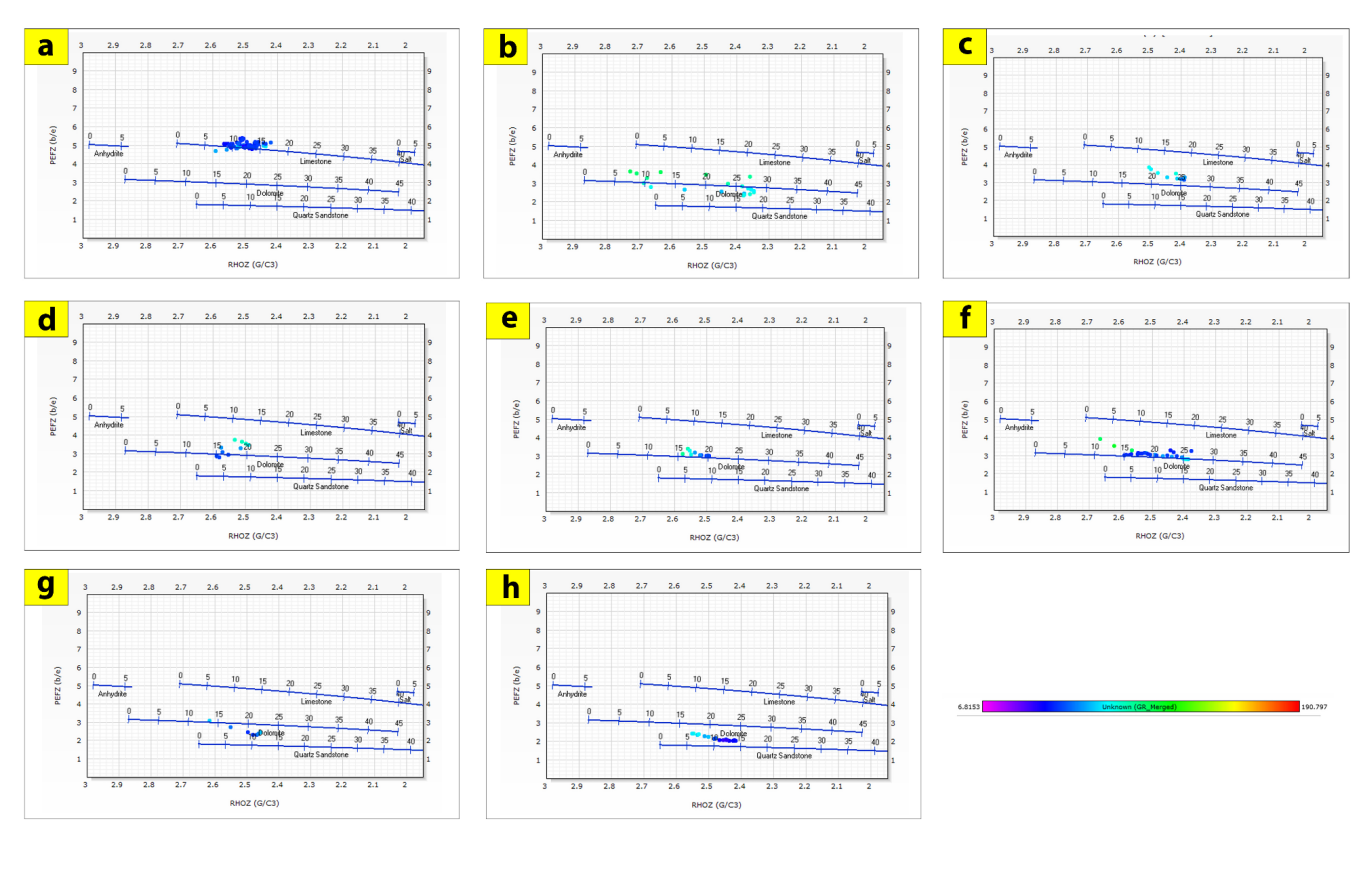
Density-PEE-Gamma Ray crossplots of the hydrocarbon-bearing zone in South Silah-1X.

**Fig. 12:**
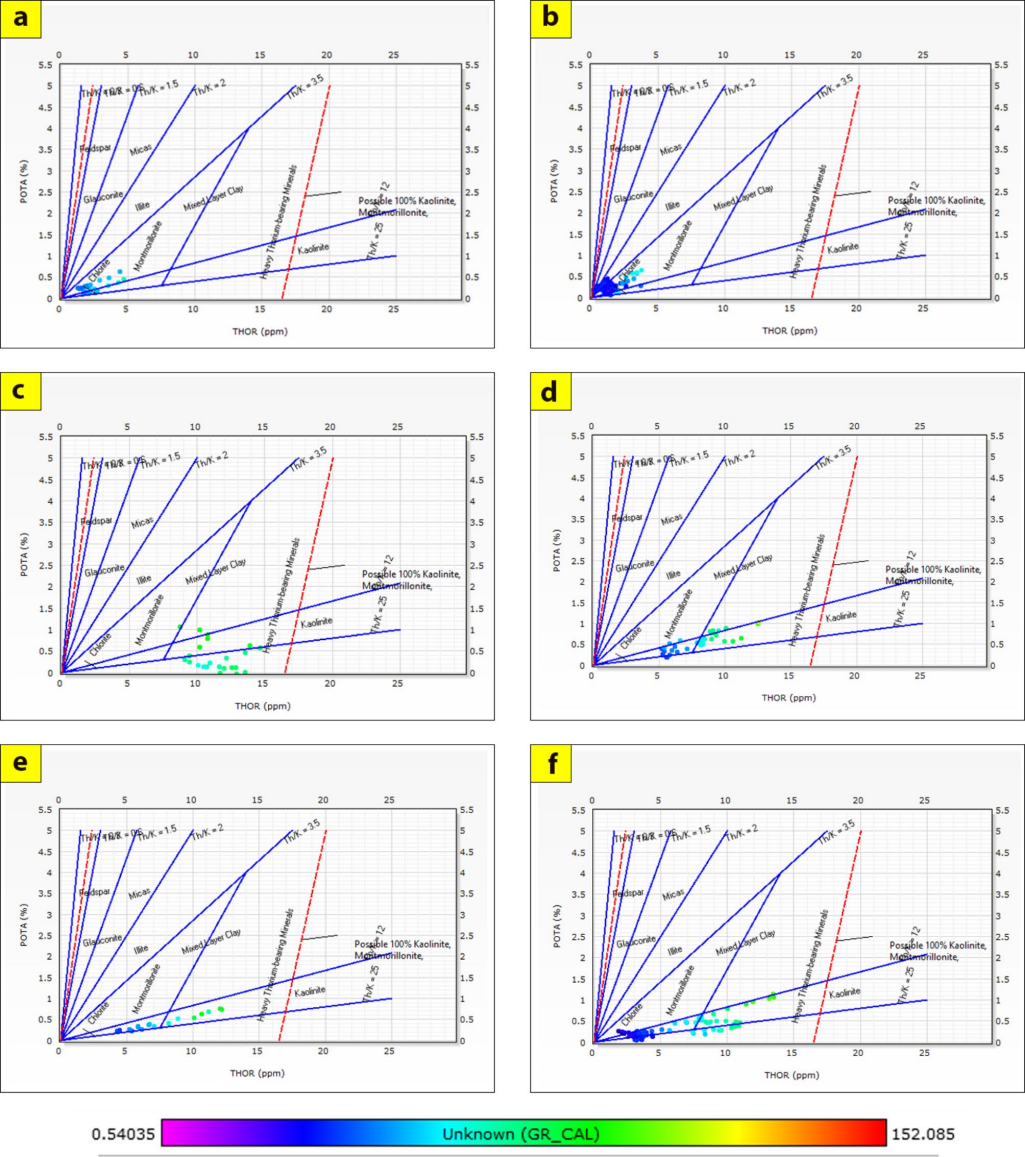
Pota-Thor-Gamma Ray crossplots of the hydrocarbon-bearing zone in Silah-15X.

**Fig. 13:**
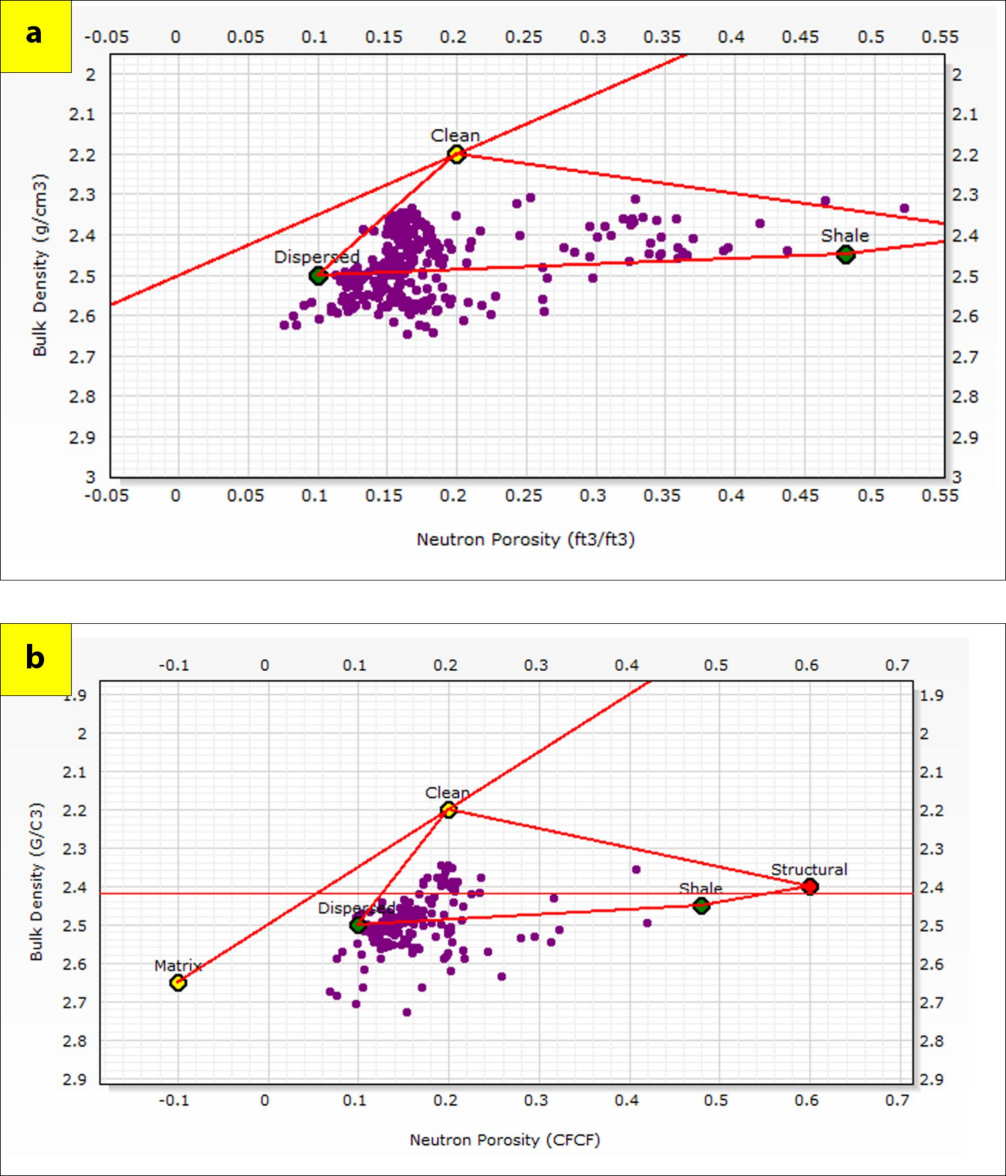
Neutron-density (Thomas-timber) plot of the hydrocarbon-bearing zones in Silah-15 (**a**), and South Silah-1X (**b**).

**Fig. 14:**
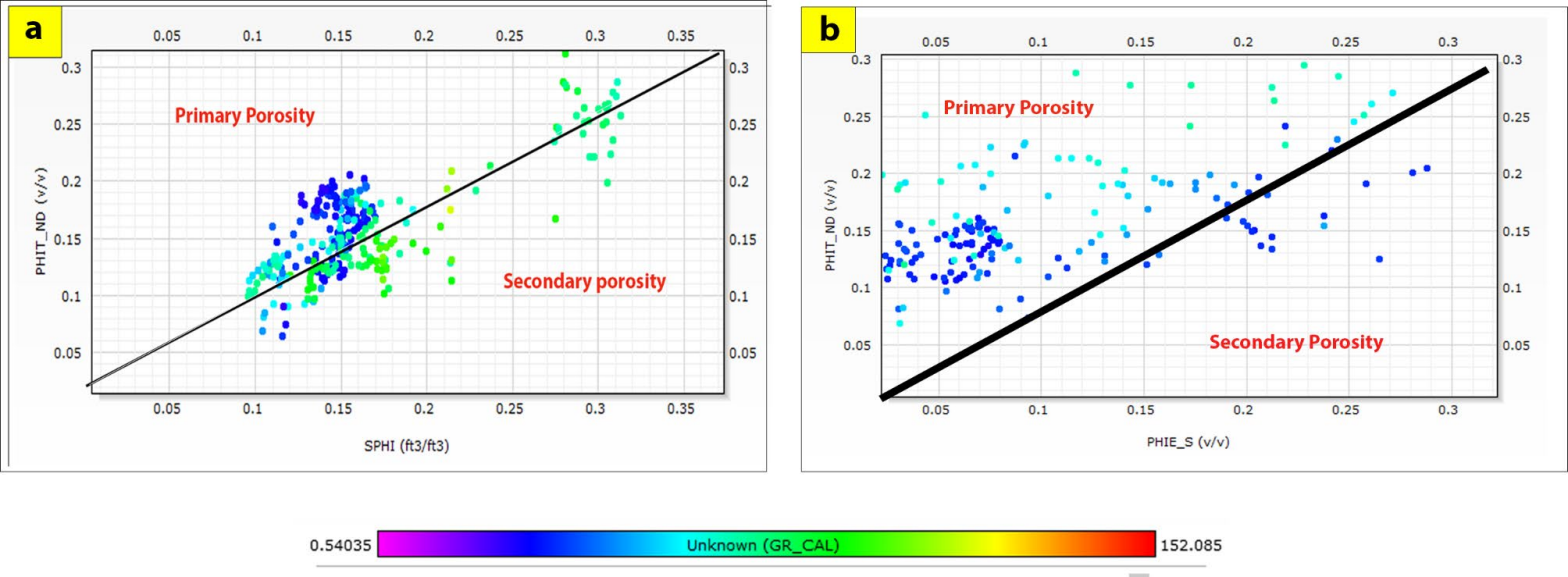
Total Porosity-Sonic Porosity plot of the identified hydrocarbon-bearing zones in Silah-15 (**a**), and South Silah-1X (**b**).

The cross-plots generated for these reservoirs through the Silah-15 and South Silah-1X drilled wells reveal that the dominant lithology ranges from shaley sandstone to sandstone and from shaley limestone to limestone with low gamma-ray values (Figs. 8, 9, 10 and 11). They have low gamma-ray responses and porosities ranging from 12 to 19%. According to the Pota–Thor cross-plots, the shale type is mainly kaolinite for the sandstone facies and chlorite for the limestone facies (Figs. 12, 13 and 14).

**Fig. 15:**
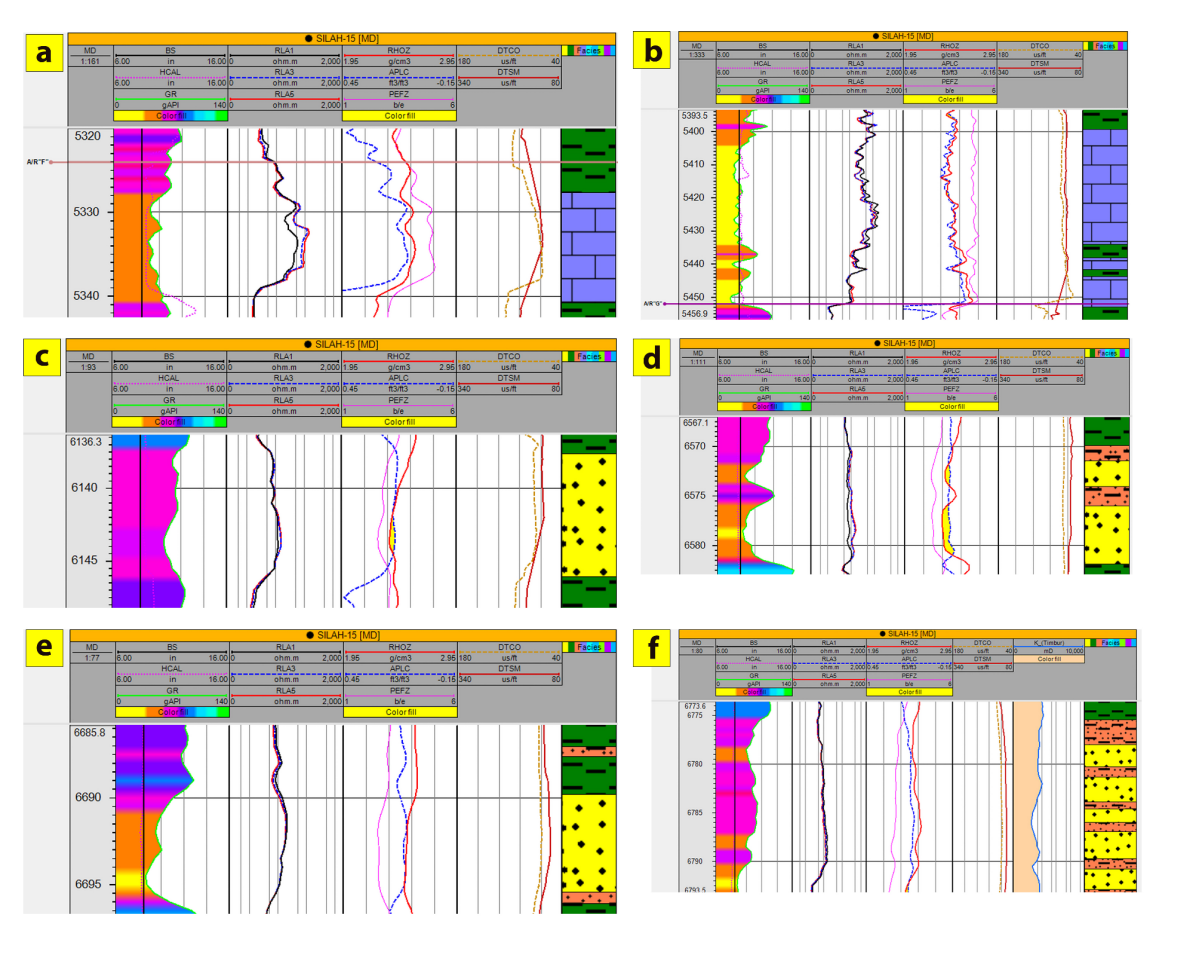
Interval depths with a composite log response and lithologic interpretation of hydrocarbon-bearing zones in Silah − 15.

**Fig. 16:**
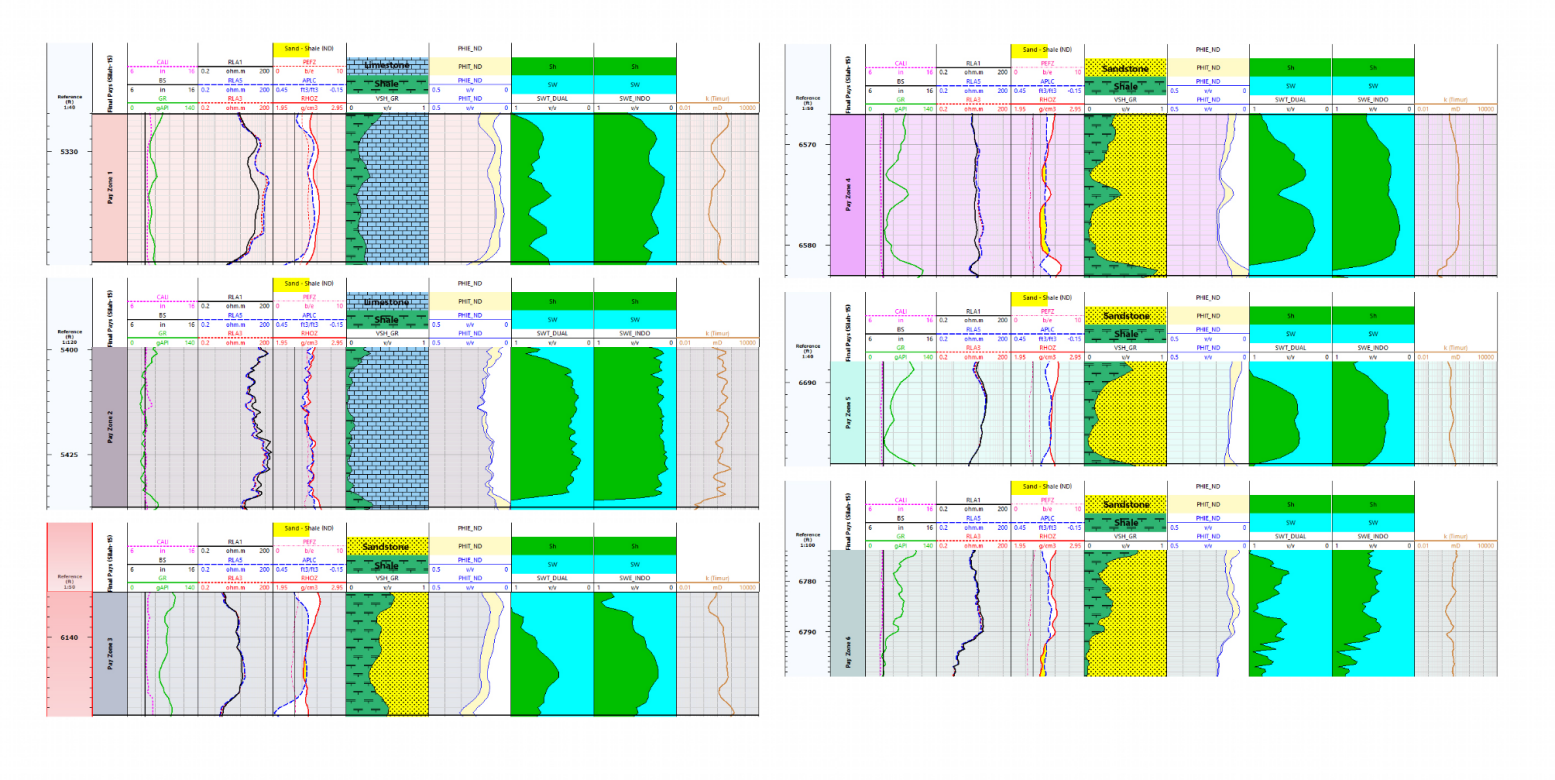
Calculated petrophysical parameters along the main hydrocarbon bearing zone in Silah-15.

**Fig. 17:**
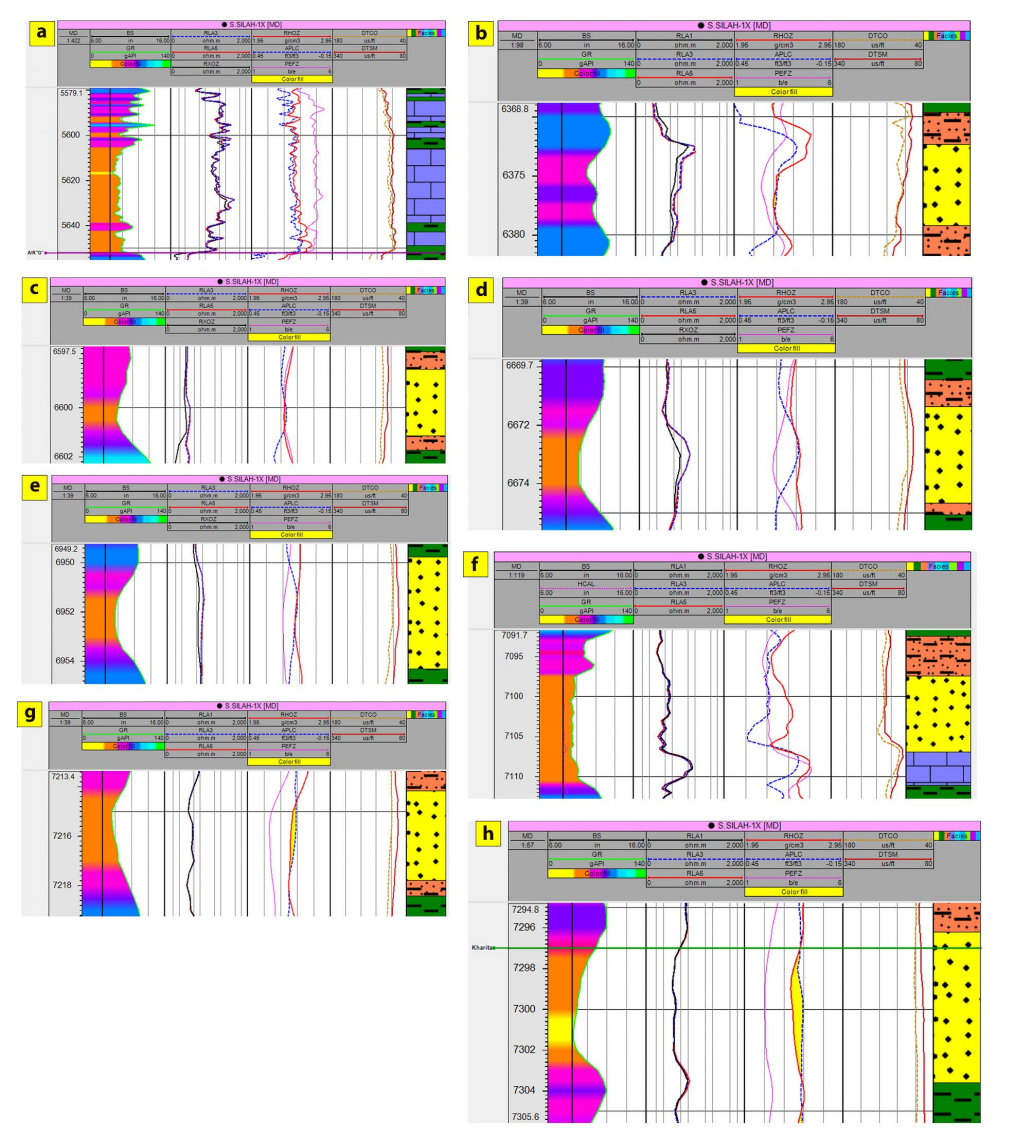
Interval depths with a composite log response and lithologic interpretation of hydrocarbon-bearing zones in South Silah-1X.

**Fig. 18:**
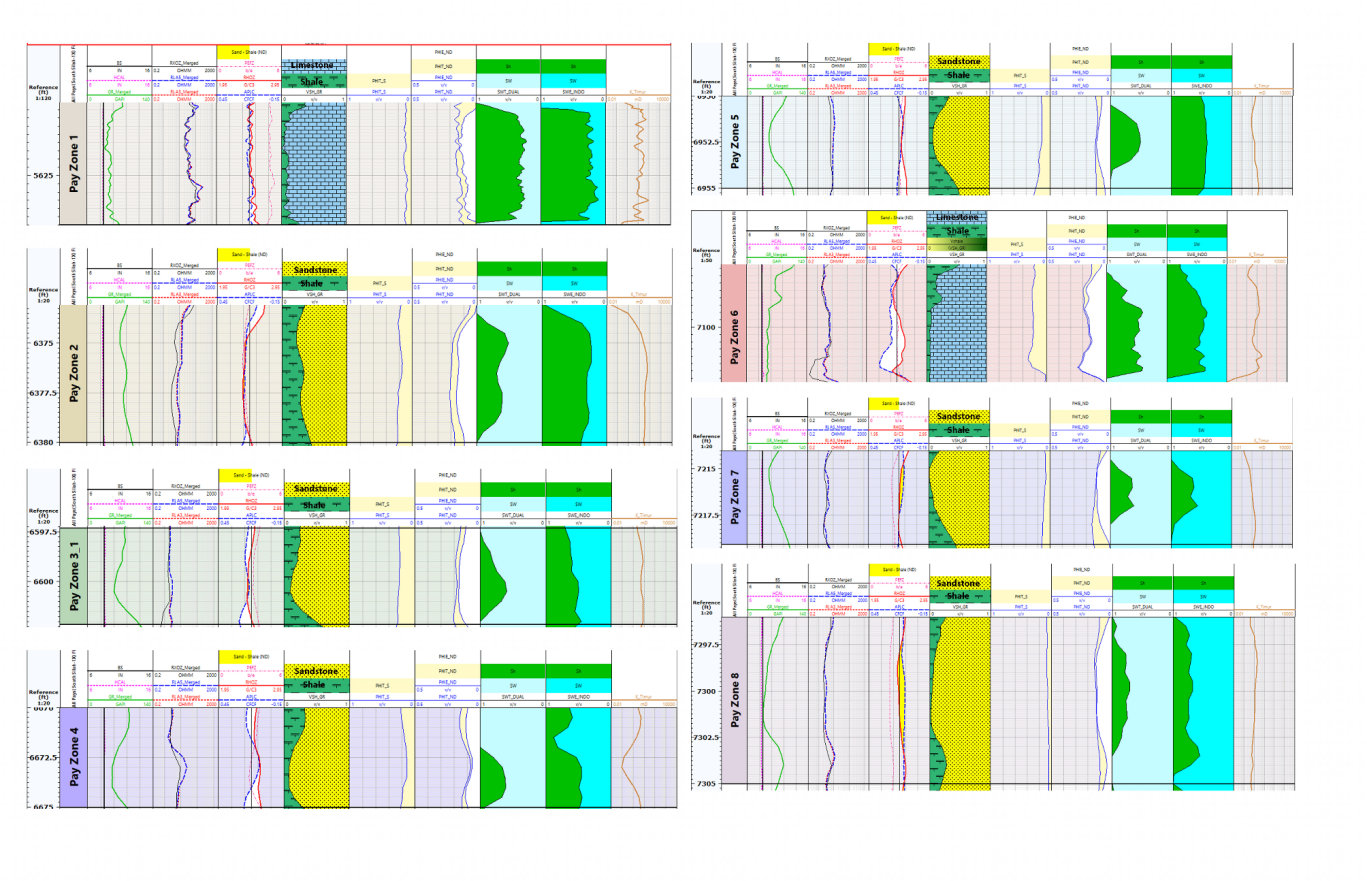
Calculated petrophysical parameters along the main hydrocarbon bearing zone of the South Silah-1X.

The interpreted results deduced from petrophysical parameters and lithologic logs reveal the presence of six and eight zones across Silah-15 (Table 2; Figs. 15 and 16) and South Silah-1X (Table 3; Figs. 17 and 18), respectively. These zones are delineated and identified as hydrocarbon-bearing reservoirs, lying within the Bahariya and Abu Roash formations, especially the G and F members (late Cretaceous).

**Table 2:** The main identified hydrocarbon-bearing reservoir zone in Silah − 15.

Zone	Interval depth (ft)		Thickness (ft)		*N*/G	Fm	Average PE (be/*n*)	Lithology
	Top	Bottom	Gross	Net				
a	5327	5339	12	11.5	0.958	A/*R*” F”	4.674	L.S
b	5400	5442	42	42	1	A/R” F”	4.767	L.S
c	6135	6147	12	5.5	0.458	A/R"G”	2.783	S.S with Shale
d	6567	6683	16	14	0.875	U. B	2.654	S.S with Siltstone
e	6688	6696	8	6	0.75	U. B	2.601	S.S with Siltstone
f	6775	6793	18	18	1	U. B	2.352	S.S with Siltstone

**Table 3:** The main identified hydrocarbon-bearing reservoir zone in South Silah-1X.

Zone	Interval depth (ft)		Thickness (ft)		*N*/G	Fm	Average PE (b/e)	Lithology
	Top	Bottom	Gross	Net				
a	5600	5640	40	38.5	0.962	A/*R* (F)	4.985	L.S
b	6370	6380	10	6	0.6	A/R (G)	2.594	S.S and Siltstone with minor L.S
c	6597	6602	5	5	1	U. B	3.39	S.S and Shale
d	6670	6675	5	3.5	0.7	U. B	3.418	S.S with Siltstone
e	6950	6955	5	5	1	U. B	3.118	S.S and Siltstone with minor L.S
f	7091	7107	16	14.5	0.906	U. B	3.029	L.S and S.S with minor shale
g	7214	7218	4	3	0.75	L.B	2.379	S.S and Siltstone
h	7296	7305	9	8.5	0.944	L.B	2.167	S.S and Siltstone

**Fig. 19:**
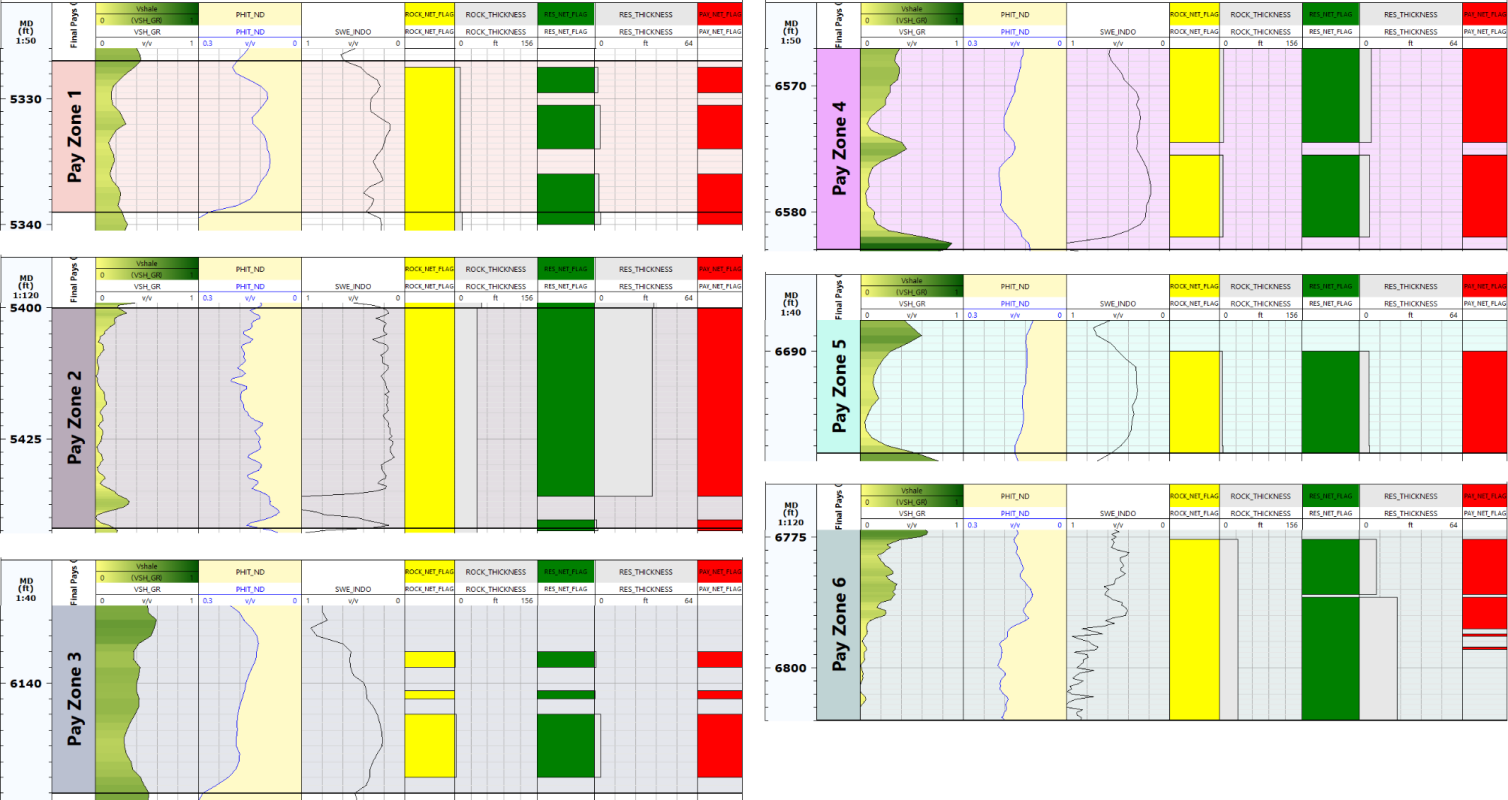
Applying hydrocarbon-bearing zone’s cutoffs along Silah-15.

The determined petrophysical parameters for the hydrocarbon-bearing zones (six zones) in Silah-15 reveal that only two permeable limestone bodies (1 and 2) are present, whereas the others are sandstone (3 to 6) in Abu Roash (F and G members) and the upper Bahariya formations (Table 4). These reservoir zones have depth intervals of 5327–6794 ft, shale volumes of 7.7 − 22%, average effective porosities of 7.8 – 13.9%, and hydrocarbon saturations of 19-62%. According to these results, the porosity values in all reservoir zones are rated moderate to good, with a low water saturation indicating a high proportion of hydrocarbons. In terms of the movability index, most of the hydrocarbon zones are moveable. The average permeability is observed to be fair to moderate, with average values ranging from 4.7 to 34.4 mD. The average BVW ranges from 0.003 to 0.096, so the sandstone bodies are characterized by fine to medium grains. The net- reservoir thickness ranges from 6 to 42 ft, but the net- pay thickness ranges from 5.5 to 37.5 ft (Fig. 19).

**Table 4:** Sum and average values of computed petrophysical parameters of the selected hydrocarbon-bearing zone in Silah-15.

Zone Name	Interval (ft)	Gross Thickness (ft)	Net Thickness (ft)	Average Total Porosity (gm/cc)	Average Effective Porosity (Φe)	Avg Vsh (Older)	Avg SW-INDO	Avg SW-DWT	Avg SXO (frac)	SW/SXO -INDO (frac)	BVW (V/V)	Avg So (frac)	Avg K Timur (mD)	Net pay (ft)
1	5327–5339	12	11.5	0.137	0.078	0.221	0.283	0.667	0.33	0.857	0.0038	0.717	18.8	8.5
2	5400–5442	42	42	0.149	0.128	0.077	0.189	0.303	0.387	0.488	0.0281	0.811	26.88	37.5
3	6135–6147	12	5.5	0.175	0.121	0.339	0.305	0.563	0.385	0.792	0.0533	0.695	34.43	5.5
4	6567–6683	16	14	0.165	0.14	0.205	0.306	0.424	0.535	0.571	0.0504	0.694	12.36	14
5	6688–6696	8	6	0.124	0.107	0.139	0.363	0.465	0.437	0.830	0.045	0.637	4.76	6
6	6773–6793	21	21	0.156	0.139	0.136	0.618	0.793	0.75	0.824	0.096	0.382	5.546	16.5

**Fig. 20:**
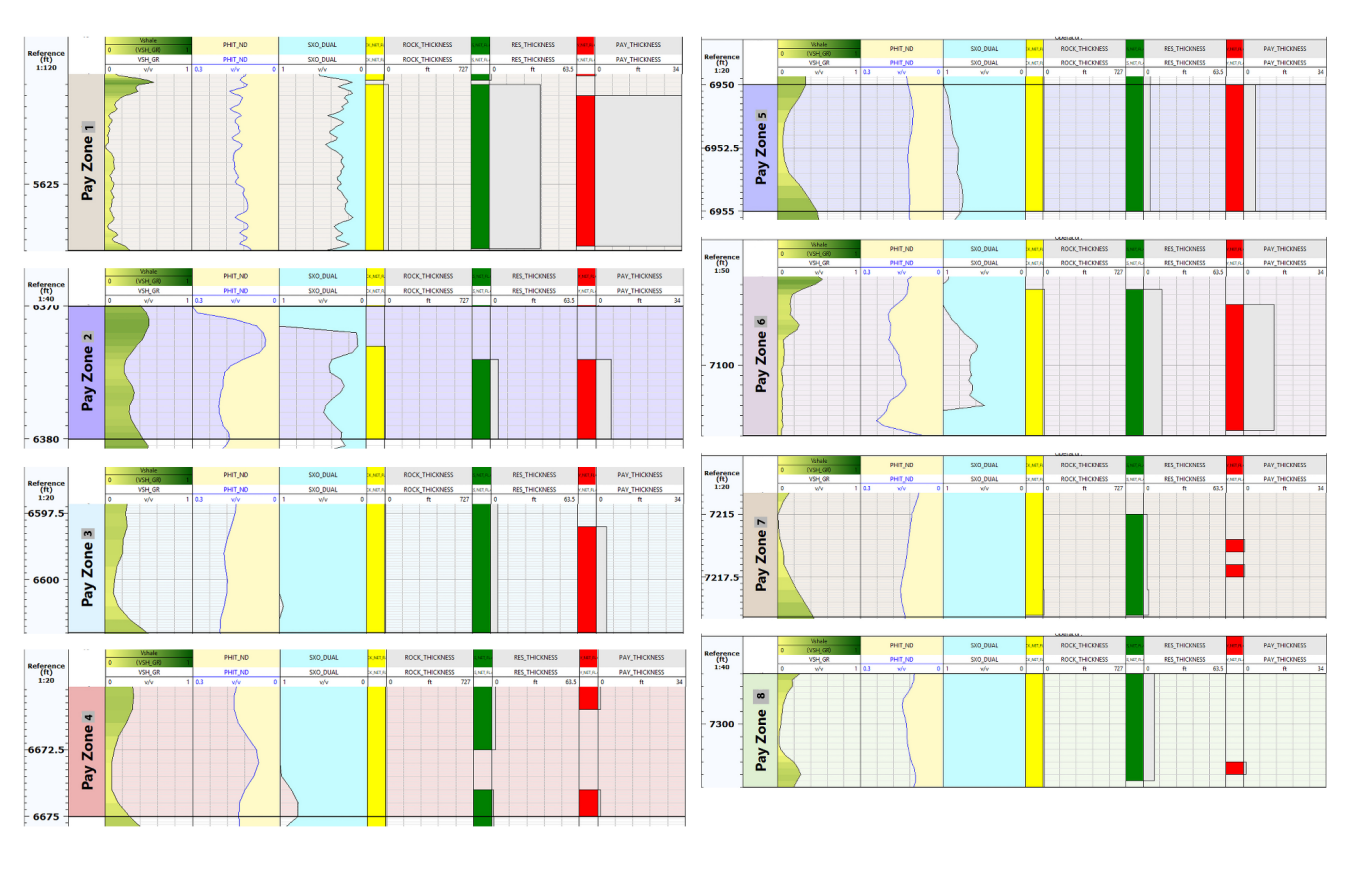
Applying hydrocarbon-bearing zone’s cutoffs along South Silah-1X.

The eight zones detected in the South Silah-1X drilled well have interval depths from 5600 ft to 7305 ft in the Abu Roash (F and G members) and Bahariya (upper and lower) Formations (Table 5). The estimated petrophysical parameters for these reservoir zones include shale volume ranging from 5.7 to 23%, average effective porosity ranging from 8 to 15%, and water saturation ranging from 33 to 73%. According to these results, the porosity values present in all reservoir zones are rated moderate to good, and low water saturation indicates a high proportion of hydrocarbons, except in Zone 8, which has high water saturation. Because the movability index is high, these zones are considered moveable for hydrocarbons. The average permeability (0. 9–31 mD) is observed to be fair to moderate. Zones 2, 3, 4, 5, 7, and 8 are shaley sandstones, but Zones 1 and 6 are shaley limestones with moderate secondary porosities (vugs and/or fractures). The net- reservoir thickness ranges from 3 to 38.5 ft, but the net- pay thickness ranges from 1 to 38.5 ft (Fig. 20). These zones have the potential for oil production, with the exception of Zone 8, which has no pay.

**Table 5:** Sum and average values of computed petrophysical parameters of the selected hydrocarbon-bearing zone in South Silah-1X.

Zone Name	Interval (ft)	Gross Thickness (ft)	Net Thickness (ft)	Average Total Porosity (gm/cc)	Average Effective Porosity (Φe)	Avg Vsh (Older)	Avg SW-INDO (frac)	Avg SW-DWT (frac)	Avg SXO (frac)	SW/SXO -INDO (frac)	BVW (V/V)	Avg So (frac)	Avg K Timur (mD)	Net pay (ft)
1	5600–5640	40	38.5	0.134	0.08	0.111	0.338	0.38	0.36	0.90	0.0452	16.9	1.8	38.5
2	6370–6380	10	6	0.187	0.138	0.293	0.366	0.661	0.377	0.777	0.0684	24.553	2.12	6
3	6597–6602	5	5	0.177	0.147	0.195	0.615	0.804	0.911	0.652	0.108	5.534	31.6	4
4	6670–6675	5	3.5	0.139	0.105	0.224	0.648	0.905	0.797	0.813	0.090	1.99	7.1	2
5	6950–6955	5	5	0.12	0.091	0.184	0.444	0.748	0.678	0.654	0.0532	1.884	10.3	5
6	7091–7107	16	14.5	0.174	0.158	0.107	0.496	0.578	0.799	0.579	0.0863	7.104	0.88	12.5
7	7214–7218	4	3	0.128	0.111	0.057	0.696	0.725	1	0.695	0.089	1.136	7.93	1
8	7296–7305	9	8.5	0.124	0.098	0.115	0.731	0.841	0.927	0.788	0.090	1.039	8.22	0

**Fig. 21:**
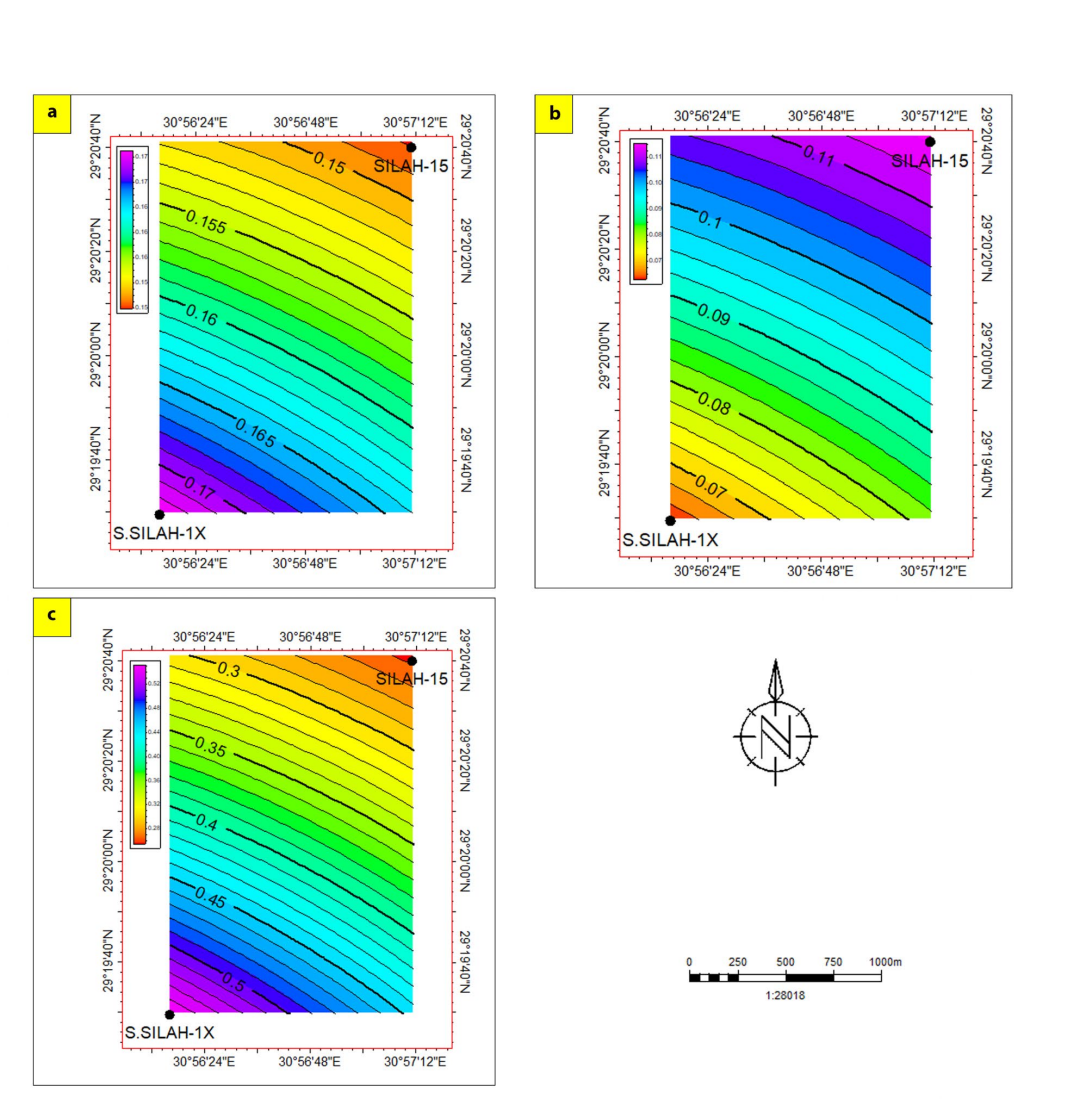
Lateral variation distribution of the estimated petrophysical parameters of AR/F zone; shale content (**a**), effective porosity (**b**), and water saturation (**c**).

**Fig. 22:**
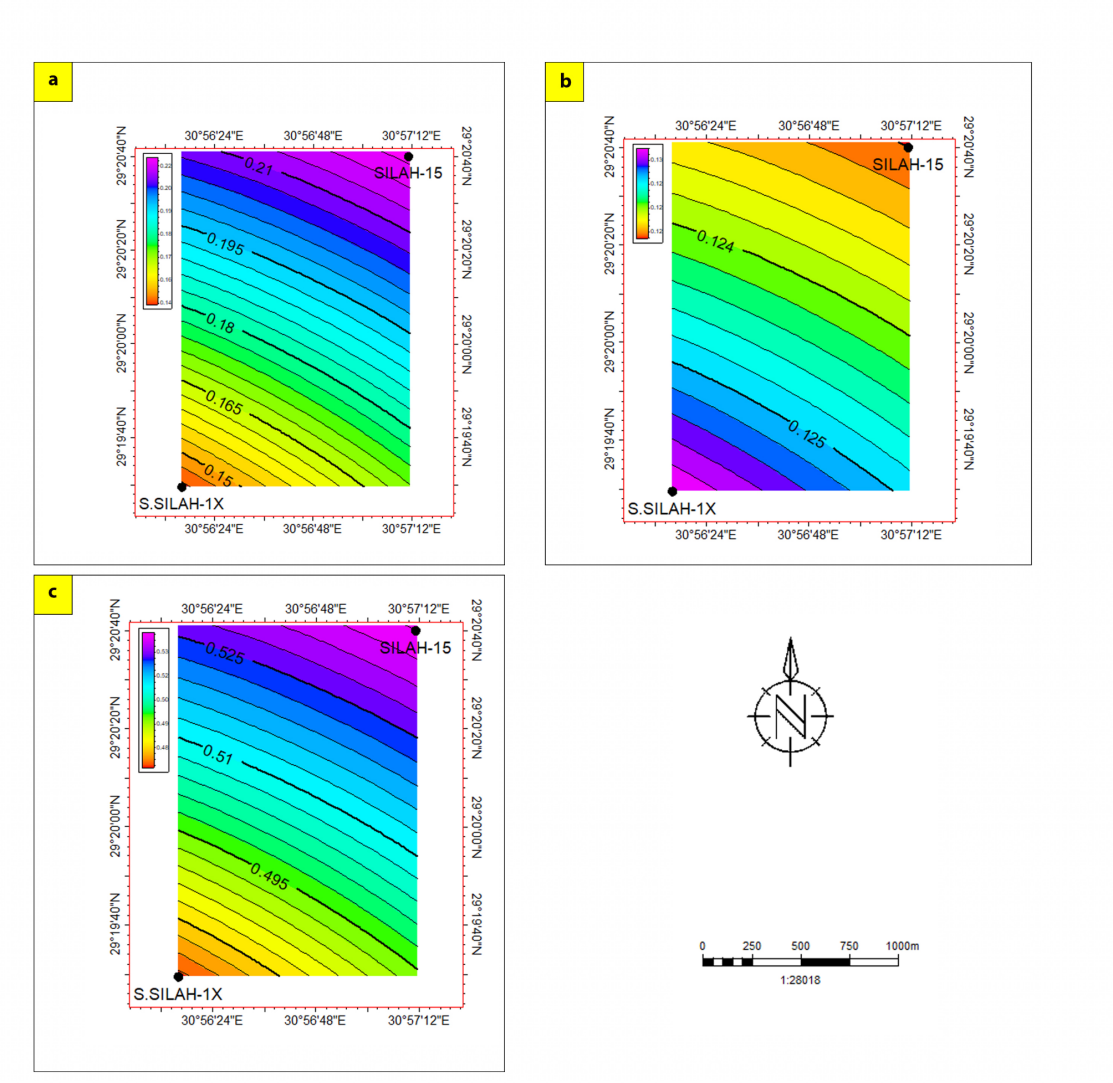
Lateral variation distribution of the estimated petrophysical parameters of upper Bahariya zone; shale content (**a**), effective porosity (**b**), and water saturation (**c**).

To assess and evaluate the hydrocarbon potential of reservoir zones (AR/F and upper Bahariya), isoperimetric maps were constructed in the Silah field (Figs. 21 and 22). These maps showed where all the known petrophysical parameters were found in relation to the different areas that might be good for looking for AR/F and UB reservoirs. Also, these maps highlighted the reservoir shale contents, effective porosity, and water saturations for the investigated wells. For contouring and interpolation, the data from each well within the net pay interval were arithmetically averaged and plotted on a map.

Relating to AR/F member of reservoir zones, shale volume (14.5- 19%) and water saturation distribution maps (30- 50%) are identified in Silah-15 (Fig. 21a and c). Lower values are recognized in the NE direction (Silah-15), while higher values are detected in the SW direction (South Silah-1X). The distribution map of effective porosity shows the lowest value (7%) in South Silah-1X while the highest value (11%) in Silah-15, so the maximum effective porosity is more pronounced in the northeastern while the minimum effective is pronounced in the southwestern parts of the study area (Fig. 21b).

Shale volume (15 − 21%), effective porosity (11- 14%), and water saturation (46- 54%) distribution through UB reservoir zones were identified (Fig. 22). The V_sh_ and SW spatial distribution indicates higher values (near Silah-15) along the northeast direction, while lower values are more pronounced along the southwest direction (near South Silah-1X). The distribution map of effective porosity shows the lowest value is detected at Silah-15 while the highest value is observed at South Silah-1X, so the maximum effective porosity is more pronounced in the southwestern while the minimum effective is pronounced in the northeastern parts of the study area (Fig. 22b).

In general, the quality of the reservoir zones of the Silah drilled wells is hardly affected by either the local or the regional geology of the area. The high shale contents in the identified Bahariya and Abu Roash Formations play important roles as source rock and seal rock above the reservoir rock. The present shale in these identified reservoirs is dispersed throughout the reservoirs and is mainly composed of kaolinite, chlorite, and montmorillonite. Locally, the Bahariya Formation is highly composed of clays with very fine grains, which tend to fill in open spaces between coarser grains (sand particles). This eventually reduces the porosity and permeability, which are the key parameters for a good reservoir rock. The Abu Roash (F) and (G) members are composed of fair to moderate secondary porosities (voids or fractures), which assist in forming a high capacity for hydrocarbon preservation.

## Conclusions

- The critical analysis of well-logging data revealed six and eight hydrocarbon potential zones across the Silah-15 and South Silah-1X drilled wells, respectively, within the Abu Roash (G and F member) and Bahariya formations in the Silah field, El-Fayoum Concession. The qualitative analyses of wireline logging data confirmed that these zones presented positive indications of potential hydrocarbon intervals. These indications include consistently low values in the gamma-ray logs (low shale oil content), a pattern of intersection between density and neutron logs (characterizing the sandstone matrix) and high true resistivity (presence of oil). The high porosity of this member may be due to a variety of factors, such as the depositional environment and compaction history. The specific factors contributing to the high porosity of the Abu Roash/F member, in this case, need to be investigated on the basis of the geological context and characteristics of the sediment. Additionally, the presence of siliceous and calcareous cements in limestone can reduce compaction and help preserve porosity. Structural features such as fracturing or vugs may have increased the porosity of the member. These factors can combine to create favourably conditions for the development of high-porosity reservoir rocks, which means that this member has the best reservoir qualities.
- The total porosities are high, reaching 18.7% in Zone 2 (South Silah-1X) and 17.55% in Zone 3 (Silah-15). The effective porosity is relatively high in all intervals, ranging from 14% in Zone 4 (Silah-15) to 15% in Zone 6 (South Silah-1X). The lowest water saturation (18.9%) is recorded in Zone 2 (Silah-15), whereas the highest value (73%) is detected in Zone 8 (South Silah-1X). The shale volumes of these zones range from low to moderate, with values varying between 5.7% in Zone 7 (South Silah-1X) and 30.5% in Zone 3 (Silah-15). The BVW ranges from 0.0038 in Zone 1 (Silah-15X), to 0.108 in Zone 3 (South Silah-1X). The net pay thickness is 37.5 ft in Zone 2 (Silah-15) and 38.5 ft in Zone 1 (South Silah-1X). These zones are laying at depths ranging from 5400 to 5442 ft (Zone 2), and from 5600 to 5640 ft (Zone 1) within the Abu Roash/F member. These conclusions indicate a positive occurrence of hydrocarbons in Zone 2 (Silah-15) and Zone 1 (South Silah-1X), which are composed of limestone.
- Lateral variation distribution of the estimated petrophysical parameters of AR/F reservoir zone indicate that the best location for drilling and production lies in the northeast direction at Silah-15. These trends may be due to gradual rock porosity modifications throughout geological periods and the environmental factors influencing the depositional characteristics. While, this distribution pattern of Bahariya reservoir zone reveals that the area in the SW direction is the most favorable for oil accumulation.
- Drilling new potential wells is more favored within the central to southwestern part of the investigated area. This proves that the exploration activities are economically favorable for hydrocarbons in the neighborhood of South Silah-1X, which lies in the southwestern part of the studied area.
- The Abu Roash/F member has been identified in this study as an oil pay. The close agreement between the petrophysical parameters of the investigated intervals within this member and others in the Northwestern Desert suggests that there may be reservoir continuity and similarity, indicating that the member is a promising hydrocarbon reservoir in the Silah field. The shale formation lying below and above the identified reservoir zones could be interpreted as a source or seal rock.

## Data Availability

The datasets used and/or analysed during the current study available from the corresponding author on reasonable request.

## References

[1] Asquith, G. & Gibson, C. *Basic well log analysis for geologists: Methods in Exploration series* (In: AAPG. Tulsa, 1982).

[2] Ghassal, B. I. et al. & alah El Khoriby, E. Source rock potential and depositional environment of Upper Cretaceous sedimentary rocks, Abu Gharadig Basin, Western Desert, Egypt: An integrated palynological, organic and inorganic geochemical study. *Int. J. Coal Geol.* **186**, 14–40 (2018).

[3] Asquith, G. B., Krygowski, D. & Gibson, C. R. *Basic well log analysis*16305–371 (American Association of Petroleum Geologists, 2004).

[4] Ali, W. A., Deaf, A. S. & Mostafa, T. 3D geological and petrophysical modeling of Alam El-Bueib Formation using well logs and seismic data in Matruh Field, northwestern Egypt. *Sci. Rep.* **14**, 6849. 10.1038/s41598-024-56825-5 (2024). 38514735 10.1038/s41598-024-56825-5 PMC10957879

[5] Hakimi, M. H. et al. Hydraulic fracturing as unconventional production potential for the organic-rich carbonate reservoir rocks in the Abu El Gharadig Field, north western Desert (Egypt): Evidence from combined organic geochemical, petrophysical and bulk kinetics modeling results. *Fuel* **334**, 126606 (2023).

[6] Eysa, E. A., Ramadan, F. S., Nady, E., Said, N. M. & M. M. & Reservoir characterization using porosity–permeability relations and statistical analysis: A case study from North Western Desert Egypt. *Arab. J. Geosci.* **9**, 1–9. 10.1007/s12517-016-2430-x (2016).

[7] Sarhan, M. A., Basal, A. M. K. & Ibrahim, I. M. Integration of seismic interpretation and well logging analysis of Abu Roash D Member, Gindi Basin, Egypt: Implication for detecting and evaluating fractured carbonate reservoirs. *J. Afr. Earth Sc.* **135**, 1–13 (2017).

[8] Makled, W. A., Mostafa, T. F., Sawy, E., Mousa, M. Z., Ragab, M. O. & D. A. & Petroleum play of the lower cretaceous Alam El Bueib formation in the El Noor-1X well in the north Western Desert (Egypt): A sequence stratigraphic framework. *Mar. Pet. Geol.* **116**, 104287. 10.1016/j.marpetgeo.2020.104287 (2020).

[9] Deaf, A. S., Omran, A. A., El-Arab, E. S. Z. & Maky, A. B. F. Integrated organic geochemical/petrographic and well logging analyses to evaluate the hydrocarbon source rock potential of the Middle Jurassic upper Khatatba Formation in Matruh Basin, northwestern Egypt. *Mar. Pet. Geol.* **140**, 105622 (2022).

[10] Dolson, J. C. et al. The petroleum potential of Egypt. In Petroleum Provinces of the 21st Century, Memoir 74. AAPG Bulletin (ed. Morgan, W. A.) 453–482 (2001). 10.21608/jpme.2022.125833.1117

[11] Abdel-Fattah, M. I. Impact of depositional environment on petrophysical reservoir characteristics in Obaiyed Field, Western Desert, Egypt. *Arab. J. Geosci.* **8**, 9301–9314 (2015).

[12] Elhossainy, M. M., Basal, A. K., ElBadrawy, H. T., Salam, S. A. & Sarhan, M. A. Well logging data interpretation for appraising the performance of Alam El-Bueib reservoir in Safir Oil Field, Shushan Basin Egypt. *J. Petrol. Expl Prod. Tech.* **11**(5), 2075–2089. 10.1007/s13202-021-01165-7 (2021).

[13] El Ghamry, M. N., Amawy, E., Hagag, W. & M., & The role of Late Cretaceous wrench tectonics in hydrocarbon endowment in El-Gindi Basin, northern Western Desert, Egypt. *Mar. Pet. Geol.* **112**, 104093 (2020).

[14] Said, R. The geology of Egypt. Rotterdam: 734 (1990).

[15] Hantar, G. North Western Desert, in Geology of Egypt (ed Said, R.) 293–327, A. A. Balkema, Rotterdam (1990).

[16] Zaid, M. S., Abd El-Rassoul, S. M. & Abdalla, I. M. Soil Limitations and land capability classification of El-Fayoum governorate. *J. Soil. Sci. Agric. Eng. Mansoura Univ.* **3**(8), 763–778 (2012).

[17] Issawi, B., Francis, M. H., Youssef, E. A. & Osman, R. A. (eds). The phanerozoic geology of Egypt: A geodynamic approach. Ministry of Petroleum and the Egyptian Mineral Resources Authority Special Publication, **81** 571 (2009).

[18] Abdel Aal, A. & Moustafa, A. R. Structural framework of the Abu Gharadig basin, Western Desert, Egypt, Proceedings of the 9th EGPC Petroleum Exploration & Production Conference,** 2** 23–50 (1988).

[19] EGPC. In: Western Desert, Oil and Gas Fields, a Comprehensive overview.11th Petroleum Exploration and Production Conference. Egyptian General Petroleum Corporation, Cairo, 1–431 (1992).

[20] Evans, A. B., Abraham, A. B. & Thompson, B. E. Integrated reservoir characterisation for petrophysical flow units evaluation and performance prediction. *Open. Chem. Eng. J.*, **13**(1) (2019).

[21] Mennan, A. Well log interpretation and 3D reservoir property modeling of Maui-B field, Taranaki Basin, New Zealand. M.S. in Petroleum Engineering, New Zealand Petroleum & Minerals, Missouri University of Science and Technology, New Zealand, 69 (2017).

[22] Larionov, V. V. Borehole radiometry. *Mosc. Nedra*. **127**, 813 (1969).

[23] Atlas, D. *Log interpretation charts: Houston*107 (Texas, Dresser Industries Inc, 1979).

[24] Poupon, A. & Leveaux, J. Evaluation of water saturation in shaly formations. In: SPWLA 12th Annual Logging Symposium. Society of Petrophysicists and Well-Log Analysts (1971).

[25] Timur, A. An investigation of permeability, porosity and residual water saturation relationships for sandstone reservoirs. SPWLA 9th Annual Logging Symposium, New Orleans, Louisiana, **4**(9) (1968).

[26] Buckles, R. S. Correlating and averaging connate water saturation data. *J. Can. Petrol. Technol.* **4**(1), 42–52 (1965).

[27] Archie, G. E. The electrical resistivity log as an aid in determining some reservoir characteristics. *Trans. AIME* **146**(1), 54–62 (1965). (1942).

[28] Abu-Hashish, M. F., Abuelhassan, M. M. & Said, A. Analysis of the Petroleum System Elements of Amana Oil Field, East Abu Gharadig Basin, Western Desert, Egypt. *J. Petroleum Min. Eng.* **24**(1). 10.21608/jpme.2022.125833.1117 (2022).

